# Cultural distortion risk and tourist loyalty at silk road heritage: The mediating roles of perceived value and satisfaction

**DOI:** 10.1371/journal.pone.0335476

**Published:** 2025-11-05

**Authors:** Kejun Wu, Lihui Su, Sen Zhang, Shuanyan Yang, Aoxue Xing, Jingbo Zhou

**Affiliations:** 1 College of Tourism, Northwest Normal University, Lanzhou, Gansu, China; 2 Gansu Tourism Development Academy, Lanzhou, Gansu, China; Xuzhou University of Technology, CHINA

## Abstract

As the core carrier of cross-cultural communication, World Cultural Heritage sites along the Silk Road face challenges from cultural distortion risk. However, the specific mechanisms linking this risk to tourist loyalty, particularly the mediating roles of perceived value and satisfaction, remain under explored. This study aims to elucidate this dynamic pathway by constructing and testing a theoretical model of “cultural distortion risk→Perceived Value→Tourist Satisfaction→Tourist loyalty “. Focusing on the Maijishan Grottoes and utilizing 381 valid questionnaires, we employed covariance-based structural equation model (CB-SEM) grounded in Knowledge-Attitude-Practice (KAP) theory and the Stimulus–Organism–Response (SOR) model reveal the chain effects of cultural distortion risk on tourists’ cognition, emotions, and loyalty. These results demonstrate: (1) cultural distortion risk significantly reduces perceived value (β = −0.409, p < 0.001), while enhancing authenticity boosts value assessment, (2) perceived value and satisfaction act as significant chain mediators between cultural distortion risk and loyalty (β = −0.397, p < 0.001), and (3) perceived value alone does not directly predict loyalty, suggesting emotional responses (satisfaction) are crucial in loyalty decisions. The study underscores the importance of cultural promotion and enhancing tourists’ cultural perception to foster satisfaction and loyalty. These findings contribute significantly to the theoretical understanding of risk perception and behavioral responses in global cultural heritage tourism, particularly by empirically validating a chain mediation mechanism. They also offer concrete, evidence-based strategies for heritage site managers to mitigate cultural distortion and enhance tourist revisit intentions.

## 1 Introduction

Cultural heritage tourism along the Silk Road confronts a sustainability crisis manifested in the tension between economic development and authenticity preservation. UNESCO’s 2023 State of the Silk Road Heritage Report documents that 67% of World Heritage sites exhibit significant cultural distortion due to commercial pressures, directly threatening their recognition as “Outstanding Universal Value” carriers under the 1972 Convention [1]. While the 2021 International Cultural Heritage Tourism Charter mandates “protection-first” principles [2], its implementation lacks evidence-based metrics to quantify how distortion risks propagate through tourist psychology – a gap impeding SDG 11.4 (strengthened heritage safeguarding) and SDG 8.9 (sustainable tourism employment). Cultural heritage is humanity’s living archive—its identity, wisdom, and civilizational DNA—demanding protection, display, and dissemination [3].

Research on Silk Road heritage tourism has achieved substantial progress in transnational coordination mechanisms, yet it critically neglects cultural ontology preservation. Regional cooperation frameworks have optimized tourism route connectivity and management systems [4,5], while recent studies establish institutional safeguards for corridor-scale conservation [6,7]. However, three fundamental gaps persist: (1) Theoretical superficiality in addressing cultural logic and authenticity dynamics, reducing heritage to commodifiable resources [8]. (2) Operational void in defining authenticity thresholds, enabling excessive commercialization that fractures cultural narratives [8]. (3) Empirical neglect of distortion risk quantification, despite its documented erosion of heritage value [6]. The ‘active utilization’ paradigm proves counterproductive, exacerbating cultural health crises that undermine SDG 11.4 targets.

Mounting market challenges in Silk Road heritage tourism underscore the urgency of this research. The heritage sites along the Silk Road face significant risks of commodification and uneven development, primarily due to fragmented cross-border management and a lack of comprehensive planning [9]. The UNESCO training manual for 2020–2021 highlighted that the increase in tourist numbers has exerted considerable pressure on these World Heritage sites [2]. The commercialization-authenticity dilemma manifests in three critical dimensions [10,11]. (1) standardized “Silk Road experiences” eroding site-specific narratives (e.g., replication of Dunhuang dance performances across unrelated sites) [12], (2) artisan crafts displaced by mass-produced souvenirs [13], and (3) ritualistic practices repackaged as theatrical shows [8]. Authenticity loss directly undermines tourists’ value perception and emotional connection, reducing revisit loyalty [14]. Crucially, while practitioners recognize authenticity’s value, they lack empirical evidence on how distortion risk quantitatively translates to loyalty erosion, hindering effective mitigation strategies [6]. This gap impedes policymakers balancing UNESCO’s “protection-first” mandate with tourism competitiveness [2].

In a tourism context, these distortions are not just internal biases but are often embedded in the destination’s environment, creating a “Cultural Distortion Risk” that tourists must navigate. This risk is not merely about physical safety but about the potential for a devalued and inauthentic experience. However, the mechanism through which cultural distortion risk affects tourist loyalty remains a significant research gap.

To address this gap, this study proposes an integrated theoretical framework based on the Stimulus-Organism-Response (SOR) paradigm [15,16] and the Knowledge, Attitude, and Practice (KAP) model. The SOR framework posits that environmental stimuli influence an individual’s internal state, which in turn drives their behavioral response [17]. In this model, cultural distortion risk acts as the negative environmental stimulus. The tourist’s internal cognitive and affective evaluations—specifically, their perceived value and satisfaction—constitute the organismic state. Finally, tourist loyalty represents the behavioral response.

This research therefore addresses three pivotal questions: (1) How does cultural distortion risk at Silk Road heritage sites directly and indirectly impact tourist loyalty through perceived value and satisfaction? (2) To what extent does cultural authenticity preservation mitigate distortion’s negative effects on perceived value? (3) What are the relative weights of cognitive versus affective mediators in loyalty formation?

To redress the paucity of causal evidence on heritage-site cultural distortion and visitor loyalty, the research adopts an integrated KAP–SOR framework and tests hypotheses with primary data collected at Maijishan Grottoes (UNESCO ID 1442). A two-stage mixed-methods design is employed: Stage 1 – Scale development and pilot using exploratory and confirmatory factor analyses to validate the cultural-distortion risk scale, perceived authenticity, PV, satisfaction, and loyalty. Stage 2 – On-site survey conducted from April–June 2024 via systematic random sampling of visitors. Structural equation modelling (PLS-SEM 4.0) is used to estimate direct, indirect, and conditional effects; bias-corrected bootstrapping assesses mediation significance. Multi-group analysis further tests moderation by authenticity preservation perceptions.

This study contributes to the field of cultural heritage tourism research by: (1) validating the cultural distortion risk scale across various contexts, which facilitates comparative analysis among different World Heritage typologies, (2) pioneering the integration of KAP and SOR frameworks, thereby elucidating the cascades of risk cognition, affect, and behavior, and (3) uncovering a sequential mediation model where distortion influences value, which in turn affects satisfaction and loyalty, with affective responses taking precedence over rational evaluations. This research establishes emotional experience as a pivotal factor in fostering loyalty and extends the applicability of the SOR framework to transcultural heritage contexts. This exploration not only enhances the theoretical comprehension of how cultural distortion risk affects tourist loyalty but also serves as a practical resource for destination managers.

## 2 Literature review and hypothesis development

### 2.1 Literature review

Cultural heritage embodies a collective treasure of exceptional universal significance, a valuable inheritance handed down from our forebears to future generations, and a unique, non-renewable asset [18]. At its core, cultural heritage tourism entails converting historical and cultural resources into sellable products to draw in visitors [19]. This field of research is one of the most vibrant within cultural heritage studies, and it is becoming more varied, especially in terms of how tourists and local communities view and value heritage sites [20]. In a study stated clearly by Gonzalez and Antonio, researchers took Venice, a water city in Italy, as the research object, relying on the vicious circle model of tourism development [21]. Furthermore, the evolution of underwater cultural heritage in Indonesia faces challenges [22], and essential oils have been shown to have a destructive effect on cultural heritage sites [23]. This suggests that, while cultural heritage is under threat, many studies have overlooked the exploration of perception and protection of these sites.

In conclusion, while previous studies have investigated the ways to protect cultural heritage, there are significant gaps in the research. This study seeks to fill those gaps by combining the KAP model with SOR theory to create a thorough theoretical framework. To overcome the limitations of existing research, this study systematically analyzes the effects of cultural heritage tourism through the lenses of cognition, emotional responses, and behavioral responses.

### 2.2 Conceptual framework

#### 2.2.1 The KAP model.

The full name of the KAP theory is the “Knowledge-Attitude-Practice”. This model gradually expanded to encompass such as environmental behavior and consumer behavior [24]. Tourism scholars examined the relationship between tourism and infectious diseases, validating the pathway of “health knowledge → attitude → behavior” [25].

Knowledge: This dimension reflects a tourist’s cognitive awareness and suspicion that the information presented is incomplete, inaccurate, or overly simplified. It is the “intellectual” component of the perceived risk. Attitude: This dimension captures the tourist’s negative affective response to perceived inauthenticity, such as feelings of disappointment or disapproval towards excessive commercialization and the staging of culture. Practice: This dimension relates to the tourist’s perception of the tangible manifestations of distortion at the site, such as observing misleading exhibits, inauthentic souvenirs, or contrived performances. It is the behavioral evidence of distortion that the tourist encounters.

#### 2.2.2 The SOR framework.

The SOR framework, originating from environmental psychology, provides a powerful lens for understanding how external factors shape consumer behavior [17]. The framework consists of three core components: Stimulus, Organism, and Response. The ‘Stimulus’ refers to external cues in the environment that affect an individual. The ‘Organism’ encompasses the internal cognitive and affective processes that are triggered by the stimulus. The ‘Response’ is the final behavioral outcome resulting from these internal processes [15].

In tourism research, the SOR framework has been widely applied to explain how destination attributes influence tourists’ internal states like emotions, satisfaction, and PV, which in turn determine their behavioral intentions such as loyalty [16,26]. For instance, studies have identified destination environment, food experiences, and service quality as stimuli that shape loyalty through the mediation of satisfaction and emotional connection [27,28].

This study adapts the SOR framework to the context of heritage tourism by conceptualizing the perceived risk of cultural distortion as a critical, yet negative, environmental stimulus. This moves beyond traditional stimuli like physical environment or service quality to include a more abstract, cognitive risk.

This study operationalizes: cultural distortion risk as a negative knowledge input triggering tourists’ authenticity cognition (e.g., awareness of historical inaccuracies in Cave 76 inscriptions). Perceived value and satisfaction as the attitudinal core reflecting cognitive-to-affective transformation (e.g., diminished value perception from commercial distortion). Tourist loyalty (revisit intention) serves as the ultimate behavioral indicator in the KAP paradigm.

This establishes a direct K → A → P pathway: Knowledge = cultural distortion risk: cognitive recognition of distortion risks. Attitude = perceived value + tourist satisfaction: Affective-cognitive evaluations arising from K. Practice = tourist loyalty: Behavioral output (revisit/recommend intention).

**2.2.2.1 Cultural distortion risk as a stimulus:** Cultural heritage authenticity and integrity are crucial for meaningful visitor experiences. However, tourism pressures can distort cultural narratives through simplification, commercialization, or framing aligned with corporate/political interests over historical accuracy [29]. This constitutes “Cultural Distortion Risk”, where tourists risk consuming inauthentic representations of the culture they seek.

The concept of distortion is well-established in psychology, often referring to cognitive distortions—irrational ways of thinking that affect an individual’s perception of reality and contribute to negative emotional states [30]. While these are internal, cultural distortion risk is an external risk perceived in the environment, stemming from how the heritage site is managed and presented. It represents a failure to communicate science and history effectively, leaving the public vulnerable to misinformation [31].

Within the SOR framework, a negative stimulus, such as cultural distortion risk, is expected to trigger adverse internal responses in the organism. This establishes a direct K → A → P pathway: Knowledge = cultural distortion risk: Cognitive recognition of distortion risks. Attitude = perceived value + tourist satisfaction: Affective-cognitive evaluations arising from K. Practice = tourist loyalty: Behavioral output (revisit/recommend intention). Cultural distortion risk as a negative knowledge input (KAP-K/ SOR-S) triggers organismic states (KAP-A/perceived value/tourist satisfaction), culminating in behavioral responses (KAP-P/ tourist loyalty).

**2.2.2.2 Perceived value and tourist satisfaction as organismic states:** The ‘Organism’ component of the SOR model represents the internal processing of external stimuli. This study focused on two key organismic variables: Perceived Value and Tourist Satisfaction.

Perceived Value is a tourist’s overall assessment of the benefits received from a tourism experience relative to the costs (monetary, time, and effort) [32]. It is a multidimensional construct that can include functional, emotional, social, and epistemic (knowledge-seeking) benefits. At a heritage site, value is derived not just from aesthetics but from the sense of connection to history, learning, and existential authenticity [16]. When cultural distortion risk is high, the epistemic and emotional value of the experience is likely to be severely compromised, as the authenticity that underpins these benefits is questioned.

Tourist Satisfaction is an affective state resulting from the comparison of pre-visit expectations with the actual performance of the destination [27]. It is a crucial mediator between destination attributes and behavioral intentions [33]. A satisfactory experience at a heritage site often depends on feeling an authentic connection and gaining meaningful insights [15].

The relationship between perceived value and tourist satisfaction is well-documented, with perceived value generally considered an antecedent to satisfaction [34]. When tourists perceive that they have received high value for their investment of time and money, they are more likely to feel satisfied with their overall experience.

**2.2.2.3 Tourist loyalty as the response:** Tourist Loyalty is the ultimate ‘Response’ in our SOR model. It is a deeply held commitment to re-patronize a preferred destination consistently in the future, despite situational influences and marketing efforts having the potential to cause switching behavior [35]. It is typically measured through two key dimensions: conative loyalty and word-of-mouth [36].

Both perceived value and satisfaction are established as strong direct predictors of tourist loyalty [32,33]. Tourists who feel they have received a valuable experience and are satisfied with their visit are naturally more inclined to return and share their positive experiences with others [34].

**2.2.2.4 The mediating roles of perceived value and tourist satisfaction:** Based on the SOR logic, the core of the model lies in the mediating function of the organismic variables. This study argues that the negative impact of cultural distortion risk on tourist loyalty is not direct but is channeled through the tourist’s internal evaluations. When tourists perceive cultural distortion, it first lowers their PV of the experience and their satisfaction, which in turn leads to a decreased likelihood of demonstrating loyalty.

Several studies in tourism have confirmed similar mediation pathways. For example, destination attributes affect loyalty through the mediation of satisfaction [37], and memorable experiences influence loyalty through the mediation of memorability and aesthetics [38]. Given the established sequence from value to satisfaction, it is plausible to expect a serial mediation chain: cultural distortion risk impacts perceived value, which then impacts satisfaction, which finally impacts loyalty.

### 2.3 Hypotheses development

#### 2.3.1 Cultural distortion risk → Perceived value (h1).

Cultural distortion undermines heritage authenticity by simplifying cultural expressions for commercial purposes [11], directly reducing tourists’ value perception [39]. Cultural distortion—stemming from modernization, globalization, and inadequate conservation—erodes the authenticity of heritage sites, thereby diminishing public cultural identification [40] and support [41]. Perceived value is a pivotal driver of heritage preservation [42]; unmitigated distortion precipitates a decline in perceived value [43], which in turn reduces community engagement and conservation backing [44]. Cross-cultural variations in uncertainty avoidance, individualism, and related dimensions significantly condition the perception and management of distortion risk [45] and perceived value, necessitating culturally attuned approaches [46]. Therefore, the research hypothesis is as follows:

H1: Cultural distortion risk negatively affects perceived value.

#### 2.3.2 Cultural distortion risks→ Tourist satisfaction (H1a).

Cultural distortion risk fundamentally undermines the core value proposition of World Heritage Sites. As established in heritage tourism literature, cultural distortion risk manifests through inauthentic historical representation (e.g., simplification of complex narratives for tourist consumption) [47], over-commercialization (e.g., proliferation of incongruent commercial facilities), staged authenticity (e.g., decontextualized cultural performances), and *communication failures* in interpretation (e.g., inadequate guide training or misleading signage) [48]. These distortions constitute “framing distortions” that manipulate perceptual authenticity [49] and “communication distortions” that impede knowledge transfer [11].

When tourists perceive cultural distortion risk, three critical satisfaction pathways are disrupted: (1) Authenticity Deprivation: Tourists inherently value genuine cultural encounters [11]. Commercialization and contrived representations dissociate visitors from authentic experiences, triggering negative appraisals [50]. (2) Emotional Disconnection: Distorted cultural narratives inhibit affective bonding with the heritage setting, directly reducing enjoyment [51]. (3) Educational Value Erosion: Trivialization or misinterpretation of cultural practices compromises pedagogical efficacy, propagating misinformation and frustration [52]. While extant studies emphasize positive drivers of satisfaction (e.g., service quality [53], the risk-based perspective on cultural distortion risk’s detrimental effects remains empirically underdeveloped. The research hypothesis is as follows:

H1a: Cultural distortion risk exerts a significant negative effect on tourist satisfaction.

#### 2.3.3 Cultural distortion risks → Tourist loyalty (H1b).

Cultural distortion risk encompasses the potential and realized alterations, simplifications, commodification, or misrepresentations of authentic cultural heritage elements (tangible and intangible) driven by economic pressures, the demands of the tourist gaze, political agendas, or technological mediation [29]. Such distortion fundamentally undermines the perceived authenticity of the visitor experience [54].

When tourists encounter cultural distortion risk, it triggers significant negative psychological responses. Cognitive dissonance arises from the conflict between expectations of authenticity and the perceived inauthenticity or superficiality of the distorted presentation [54]. Furthermore, cultural distortion risk contributes to memory distortion, where simplified or stereotyped representations become the dominant recollection, hindering nuanced understanding [29]. Crucially, these cognitive processes culminate in profound emotional dissatisfaction and cynicism, analogous to negative affect generated by exposure to unrealistic cultural ideals [55].

This dissatisfaction and erosion of trust directly attack the core pillars of tourist loyalty. Dissatisfied tourists are demonstrably less likely to revisit a destination or recommend it to others [56]. Cultural distortion risk is perceived as a form of deception, damaging the destination’s image and eroding the trust essential for a long-term relationship [11]. Consequently, cultural distortion risk is hypothesized to lead to reduced re-visitation intentions and negative word-of-mouth communication, thereby exerting a significant negative effect on overall tourist loyalty. Hence:

H1b: Cultural distortion risk negatively affects tourist loyalty.

#### 2.3.4 Perceived value→ Tourist satisfaction (H2a).

Based on the theoretical framework of consumer value theory and empirical evidence synthesized from recent literature, this study formally proposed Hypothesis H2a regarding the direct positive effect of perceived value on tourist satisfaction in the context of Silk Road heritage tourism, specifically at the Maijishan Grottoes.

H2a: Perceived value positively affects tourist satisfaction.

This hypothesis is grounded in the multidimensional nature of perceived value in cultural heritage settings, encompassing functional (e.g., accessibility, facilities), emotional (e.g., awe, connection), social (e.g., self-enhancement), educational (e.g., learning), and economic (e.g., value-for-money) dimensions [57,58]. Empirical studies consistently demonstrate that perceived value acts as a primary antecedent of satisfaction, as it represents tourists’ holistic assessment of benefits relative to sacrifices [32]. In heritage contexts, the authenticity and depth of cultural experiences amplify this relationship: when tourists derive high educational value (e.g., understanding historical narratives) or emotional value (e.g., spiritual resonance) from their visit, their fulfillment of intrinsic motivations elevates satisfaction [16,33]. This aligns with the SOR paradigm, where perceived value (Stimulus) triggers affective evaluations (Organism), resulting in tourist satisfaction (Response). Critically, even if peripheral services (e.g., amenities) are suboptimal, the core heritage value—rooted in authentic and meaningful engagement—can sustain satisfaction by fulfilling tourists’ fundamental quest for enrichment [47]. Thus, this study posited that enhancing multidimensional perceived value at Maijishan will directly elevate tourists’ post-visit satisfaction.

#### 2.3.5 Perceived value → Tourist loyalty (H2b).

While satisfaction is a key driver of loyalty, a growing body of research suggests that perceived value can also exert a direct influence on loyalty intentions [34]. This direct path implies that the value proposition itself can create a strong bond with the consumer, fostering loyalty even without being fully mediated by a general satisfaction judgment. In a heritage context, the unique, authentic, and profound value derived from the experience (e.g., a deep emotional connection or a transformative learning experience) may be so powerful that it directly cultivates a desire to return and recommend the site. Several studies have found empirical support for this direct link. For instance, research on TV travel products [42], industrial heritage sites [33], and general destination tourism [32] all report a significant positive relationship between perceived value and tourist loyalty. This suggests that value is not just an input for satisfaction but a foundational element for building lasting visitor relationships. Accordingly, this study hypothesized:

H2b: Perceived value positively affects tourist loyalty.

#### 2.3.6 Tourist satisfaction →Tourist loyalty (H3a).

Based on the robust theoretical foundation and extensive empirical evidence synthesized from recent literature, we formally propose Hypothesis H3a regarding the direct positive effect of tourist satisfaction on tourist loyalty in the context of Silk Road heritage tourism, specifically at the Maijishan Grottoes:

H3a: Tourist satisfaction positively affects tourist loyalty.

This hypothesis is anchored in Oliver’s Expectation-Confirmation Theory, which posits that satisfaction arises when experiences meet or exceed pre-visit expectations, thereby fostering positive behavioral intentions toward future engagement [59,60]. In cultural heritage tourism, this relationship is particularly salient due to the experience-intensive and meaning-laden nature of heritage consumption. Recent empirical studies robustly validate this linkage: Qiu (2024) demonstrated that satisfaction acts as a critical mediator between perceived value and tourist loyalty at industrial heritage sites, confirming its pivotal role in loyalty formation [33]. Fu (2023) identified a significant positive association between satisfaction and revisit intention at the Archaeological Ruins of Liangzhu City, highlighting satisfaction as a direct driver of behavioral loyalty [61]. Dai (2023) established that tourist satisfaction not only directly enhances loyalty in “red tourism” but also mediates the impact of motivation on loyalty, reinforcing its centrality in heritage contexts [15]. Wang (2023)’s meta-analysis of 242 studies identified satisfaction as one of the five most critical antecedents of loyalty across diverse tourism settings, providing overwhelming empirical weight [34]. Carvache‐Franco (2024) further corroborated this in religious tourism, where satisfaction directly strengthened loyalty toward both monuments and destination cities [61].

The mechanism underpinning H3a is clear: satisfaction derived from intellectual stimulation (e.g., understanding historical narratives), emotional resonance (e.g., awe at cultural grandeur), or aesthetic appreciation (e.g., artistic value) cultivates attitudinal commitment (preference, advocacy) and behavioral intent (revisit willingness) [53]. At Maijishan, where authenticity and cultural depth are paramount, satisfaction generated by fulfilling tourists’ quest for meaningful engagement is posited to directly translate into enduring loyalty.

#### 2.3.7 The chain mediation: Cultural distortion risk →Perceived value → Tourist satisfaction →Tourist loyalty (H4).

Based on the theoretical foundation of the SOR framework and emerging empirical evidence from heritage tourism research, we formally propose Hypothesis H4 regarding the serial mediation pathway linking cultural distortion risk to tourist loyalty through perceived value and tourist satisfaction at the Maijishan Grottoes:

H4: Perceived value and satisfaction serially mediate the relationship between cultural distortion risk and tourist loyalty.

This hypothesis posits a sequential cognitive-affective-behavioral mechanism: **S**timulus: Perceived cultural distortion risk (e.g., commodification, staged authenticity, narrative fragmentation) acts as a negative external stimulus [29]. Organism: Cultural distortion risk first diminishes tourists’ cognitive evaluation of the experience’s *worth* by undermining authenticity and intrinsic value [33]. Reduced perceived value then lowers affective fulfillment, as sacrifices (e.g., effort, cost) outweigh diminished benefits [57]. Response: Dissatisfaction ultimately reduces behavioral intentions, including revisit and recommendation [34].

Recent studies validate this chain: Qiu (2024) demonstrated perceived value → tourist satisfaction →tourist loyalty serial mediation at industrial heritage sites [33]. Zhu (2025) confirmed stimulus (experience design) →perceived value→ tourist satisfaction → tourist loyalty pathways in experiential tourism [16]. Jebbouri (2021) identified trust and satisfaction as sequential mediators between authenticity and loyalty [37]. Empirical parallels exist in contexts where inauthenticity reduces perceived value (e.g., emotional/educational value), triggering dissatisfaction and loyalty erosion [15,61]. Thus, this study hypothesized that cultural distortion risk’s negative impact on loyalty is indirect and fully transmitted through this cognitive-affective cascade.

**Fig 1:**
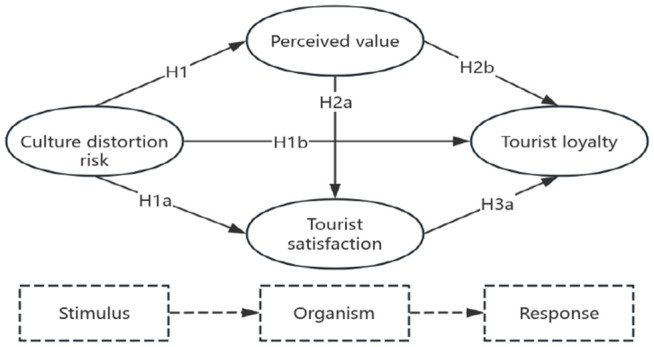
Research model.

The conceptual model for this study is presented in Fig 1.

## 3 Methodology

The study adopts a quantitative, cross-sectional survey design, employing CB-SEM to test the hypothesised causal chain. CB-SEM was selected because it allows confirmatory testing of a theory-driven model, simultaneous estimation of measurement and structural components, and unbiased parameter estimates for reflective constructs and serial mediation.

This study’s predefined causal chain—“cultural distortion risk → perceived value →tourist satisfaction →tourist loyalty “—in cultural heritage tourism constitutes a theory-driven confirmatory analysis, fully aligned with CB-SEM’s confirmatory orientation. CB-SEM demonstrates superior advantages in theoretical verification, unbiased estimation, and model fit indices, rendering it ideal for reflective measurement models and complex mediation testing. CB-SEM typically requires a minimum sample size of 200, with complex models recommending 10–20 times the number of estimated parameters. This study involves four latent variables and 12 paths, yielding approximately 20 parameter estimates. With a sample size of 381 (19 times the parameter count, well above critical thresholds), the design adheres to methodological standards while ensuring conclusion reliability and generalizability.

### 3.1 Research context

This study selects the Maijishan Grottoes, a World Cultural Heritage site situated along the Silk Road, as the case study area. The Maijishan Grottoes were inscribed on the World Cultural Heritage List on June 22, 2014, and recognized as a national AAAAA attraction in January 2011. Tianshui, the birthplace of Fu Xi and Nüwa, serves as a marvellous tourist destination [62]. The increasing number of tourists visiting the Maijishan Grottoes has impacted the preservation and inheritance of tourism resources in the scenic area. Concurrently, aggressive solicitation by souvenir vendors at the Maijishan site precinct—often involving tourist deception—prevails. Such excessive commercialization not only disrupts the sacred serenity expected of Buddhist sanctuaries but also reduces grotto culture to reductionist commodification, distorting tourists’ cognition of Maijishan’s cultural value. Following the 734 CE earthquake, a cliff collapse obliterated the central cave cluster at Maijishan Grottoes, with most surviving sculptures representing later restorations. For instance, the original inscription in Cave 76 has been obscured by whitewash, creating documented contradictions between its content and the statues’ dating. This irreversible distortion stems from compounded physical damage and restoration interventions—a prevalent risk across cave heritage sites where material deterioration induces cultural inauthenticity. Collectively, these three categories of cultural distortion risk—ontological, representational, and constructive—constitute the focal exogenous construct in our conceptual model and are measured using the newly developed Maijishan Cultural Distortion Risk Scale.

### 3.2 Research instrument

This study employed a questionnaire survey method for data collection, which is structured into two distinct parts. The first part encompasses two second-order latent variables: cultural distortion risk and perceived value, alongside two first-order latent variables: satisfaction and loyalty. Part 2 mainly collects demographic information from interlocutors, including gender, age, education, employment, monthly income, etc. The measurement scales employed in the questionnaire were derived from established instruments documented in prior literature (see Table 1), with subsequent refinements implemented to align with the specific objectives and cultural framework of this investigation. The risk of cultural distortion includes three aspects: cultural ontological distortion, cultural representative distortion, and cultural distortion risk [29]. Perceived value encompasses four dimensions: quality value, emotional value, price value, and novelty value [63], each dimension containing four items. Tourist satisfaction and loyalty consist of four and three items, respectively [64].

**Table 1:** Measurement items and sources of the questionnaire.

Variable	Dimension	Item	Source
Cultural distortion risk	Cultural ontology distortion	Address the challenge of identifying cultural uniqueness	Zhang S. N., et al. (2024)
		The difficulty of gaining cultural knowledge	
		The blurred cultural perception	
		The weak cultural impression	
	Cultural representation distortion	The presentation doesn’t match the original historical style	
		The disconnect between the presentation and the essence of local culture	
		The lack of cultural presentation	
		Cultural products may be cookie−cutter	
	Cultural constructive distortion	The original culture will deteriorate	
		The customs and habits will be lost	
		The cultural heritage will be lost	
		Be deviating from the core local culture	
Perceived value	Quality vlaue	Maintained uniform quality standards across multiple iterations	Williams P and Soutar G N. (2009) [63]
		The scenic area is well done	
		Met or exceeded industry-recognized quality thresholds	
		The efficiency of tourism services is high	
	Emotional value	A sustained state of psychological fulfillment and physiological comfort	
		Stimulated a sense of exhilaration	
		Made me related	
		Made me feel happy	
	Price value	Delivered equitable returns on monetary investment relative	
		Optimal alignment with market expectations for comparable service tiers	
		Good one for the price paid	
		The pricing structure adheres to affordability thresholds	
	Novelty value	Stimulated my sense of exploration and discovery	
		Addressed my desire for novel cultural encounters	
		Provided genuine immersion in local traditions	
		Enabled comprehensive engagement with diverse aspects of the destination’s cultural	
Tourist satisfaction		Generally satisfied	Lee et al (2016) [18]; Lu et al (2015) [64]
		All expectations are fulfilled	
		Have fun	
		Time and money spent are satisfied	
Tourist loyalty		Stay here again	Kolar and Zabkar (2010) [65]; Yi et al (2017) [66]
		Others are recommended to travel here	
		Share travel updates via social media (WeChat, QQ, etc.)	

Although the original cultural distortion risk scale used a 7-point format [29], all other constructs were validated on 5-point scales [18,33,42,57,63]. To prevent commingling phenomena, the principle of cultural-geographical adaptation is rigorously followed. All measurement scales employ a five-point Likert rating scale, which includes the options “completely disagree,” “disagree,” “neutral,” “agree,” and “strongly agree.” All scales were derived from international literature and were translated by professional translators to validate the precision of the questionnaire.

### 3.3 Data collection

Data collection occurred from March to April 2025 using a mixed-methods approach. Primary on-site surveys (n = 350 valid) were administered at the Maijishan Grottoes exit by trained researchers, with self-completed questionnaires. Supplementary online surveys (n = 35 valid) were distributed via Wenjuanxing platform. After excluding 9 invalid responses (4 offline; 5 online due to short completion times), 381 valid questionnaires were retained (95.25% response rate).

To ensure participants met study criteria, a three-step screening protocol was implemented: (1) Pre-survey Filtering: Offline: Researchers approached tourists exiting the Maijishan Grottoes Scenic Area (MGSA). Eligibility required: Age ≥ 18 years, Completion of full site visit, and Non-staff visitor status. Online: The questionnaire header explicitly stated: *“This survey is exclusively for tourists who visited MGSA within the past 3 months”*. (2) Screening Questions: Item 1: *”Did you complete your visit to MGSA today/within the last 3 months?”* (Yes/No). Item 2: *“Is this your first visit to MGSA?”* (Validation for novelty effect control) *(Respondents answering “No” to Item 1 were excluded)*.

To minimize sampling and response biases, this study adopts the following measures (Table 2):

**Table 2:** Measures to mitigate biases in the process of questionnaire data collection.

Bias Type	Mitigation Measure	Implementation
**Selection Bias**	Stratified time-slot sampling	Offline surveys conducted across 8 time slots (weekdays/weekends, AM/PM)
**Non-response Bias**	Incentive equivalence	All participants entered equal lottery for 1 × ¥5 gift cards regardless of method
**Social Desirability**	Anonymity assurance	No personal identifiers collected; cover letter emphasized academic-only use
**Measurement Bias**	Blind data entry	Undergraduates performed data entry without access to research hypotheses
**Instrument Bias**	Back-translation validation	Bilingual team translated survey into English/Chinese; verified via pilot test (n = 30)

Invalid questionnaire criteria: Completion time < 50% median duration (offline: < 5 min; online: < 3 min), Straight-lining patterns (≥80% identical responses), Contradictory answers to control items (e.g., *“I did not visit MGSA”* vs. site experience questions).

## 4 Results

### 4.1 Results common method bias text

**Fig 2:**
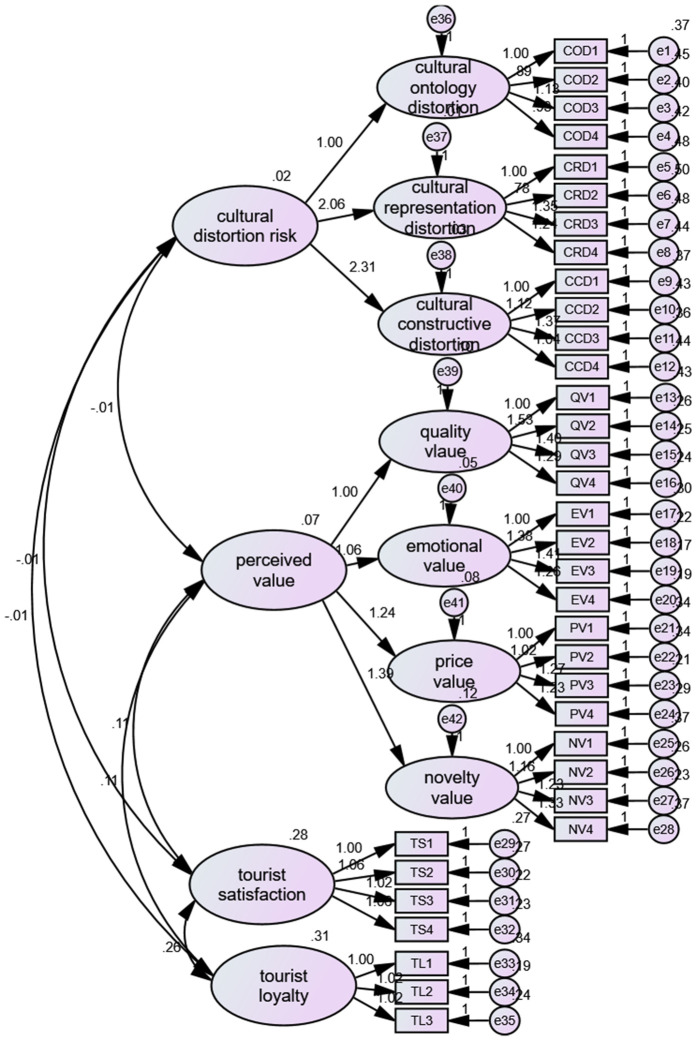
Four factor model analysis.

**Fig 3:**
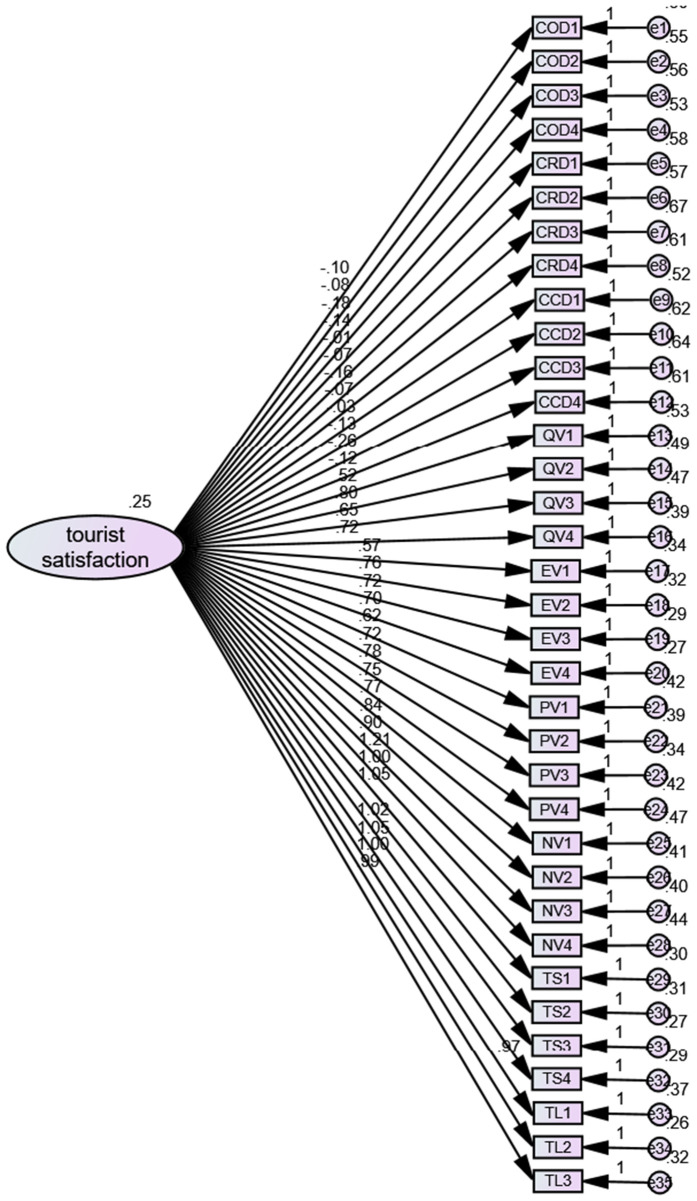
Single factor model analysis.

Given the use of self-reported data in this study, common method bias (CMB) may be a potential concern. To rigorously assess the presence of CMB, we employed a scientifically robust and stringent approach by conducting a single-factor confirmatory factor analysis (see Figs 2 and 3). Additionally, during the data collection process, we implemented several procedural remedies, such as ensuring anonymity and using both positively and negatively worded items, to mitigate the potential impact of CMB. The results of the single-factor confirmatory factor analysis indicated poor model fit, with the following fit indices: χ²/df = 3.046, CFI = 0.589, GFI = 0.668, AGFI = 0.626, NFI = 0.496, and RMSEA = 0.091 (Table 3). These findings suggest that the model does not fit the data well, which implies that CMB is not a significant issue in this study.

**Table 3:** Results common method bias text.

Model	χ2	df	χ2/df	NFI	GFI	AGFI	CFI	RMSEA
Four factor model	801.769	547	1.466	0.763	0.874	0.824	0.909	0.043
Single factor model	1705.521	520	3.046	0.496	0.668	0.626	0.589	0.091

Four factor model = CFA analysis; single factor model = tourist satisfaction.

### 4.2 Demographics of respondents

Table 4 illustrates that males constitute 41.73% and females make up 58.36% of the sample, indicating a relatively balanced gender ratio. Regarding age distribution, the majority of participants are aged between 18 and 29 years. In terms of educational attainment, individuals with an undergraduate degree represent 42.52% of the sample. Additionally, the income structure reveals that most respondents have an average monthly income exceeding 5000 yuan. Furthermore, the occupational types among respondents are distributed in a relatively balanced manner.

**Table 4:** Demographic variables descriptive statistics.

Variable	Category	Frequency	Rate (%)
Gender	male	159	41.73
	female	222	58.36
age	18-29	129	33.86
	30-39	110	28.87
	40-49	92	24.15
	50 and above	50	13.12
Education	Senior middle school and below	21	5.51
	Junior High	69	18.11
	Senior High	85	22.31
	University	162	42.52
	Master or Doctor	44	11.55
Monthly income(yuan)	3000 and below	90	23.62
	3001-5000	91	23.88
	5001-10000	143	37.53
	10001-20000	46	12.07
	200001 and above	11	2.89
Occupation	Professional	38	9.97
	Company employee	56	14.70
	Manufacturer/Technician	63	16.54
	Service employee	34	8.92
	Independent businessman	25	6.56
	Government official/Teacher	41	10.76
	Student	59	15.49
	Other	65	17.06

Reliability and Validity: To ensure convergent validity, items with factor loadings below 0.50 were removed. Post-refinement, all constructs demonstrated acceptable convergent validity: standardized factor loadings exceeded 0.50, composite reliability (CR) values were high, and average variance extracted (AVE) surpassed the 0.50 threshold. Exploratory factor analysis confirmed the scales’ structure: cultural distortion risks scale yielded three factors (“Cultural Ontology Distortion”, “Cultural Representation Distortion”, “Cultural Construction Distortion”; cumulative variance explained = 73.51%). The Perceived Value scale yielded four factors (cumulative variance explained = 68.18%), exceeding the 60% minimum standard [47].

The overall Cronbach’s alpha values for cultural distortion risk and perceived value are 0.955 and 0.926, respectively, with all variables exhibiting Cronbach’s alpha values greater than 0.70, indicating strong internal consistency within the scale. Furthermore, the CR values of all items in the scale exceed 0.7, confirming robust convergent validity among the latent variables (Table 5).

**Table 5:** Results of confirmatory factor analysis.

Item codes	Questionnaire items	SFL	α	VCC/%	AVE	CR
Cultural distortion risk						0.951
Cultural ontology distortion			0.874	72.648	0.612	0.863
COD1	Address the challenge of identifying cultural uniqueness	0.766				
COD2	The difficulty of gaining cultural knowledge	0.804				
COD3	The blurred cultural perception	0.784				
COD4	The weak cultural impression	0.775				
Cultural representation distortion			0.858	70.133	0.624	0.869
CRD1	The presentation doesn’t match the original historical style	0.753				
CRD2	The disconnect between the presentation and the essence of local culture	0.772				
CRD3	The lack of cultural presentation	0.831				
CRD4	Cultural products may be cookie−cutter	0.803				
Cultural constructive distortion			0.88	73.508	0.648	0.88
CCD1	The original culture will deteriorate	0.82				
CCD2	The customs and habits will be lost	0.791				
CCD3	The cultural heritage will be lost	0.827				
CCD4	Be deviating from the core local culture	0.781				
Perceived value						0.888
Quality value			0.858	70.045	0.608	0.86
QV1	Maintained uniform quality standards across multiple iterations	0.675				
QV2	The scenic area is well done	0.81				
QV3	Met or exceeded industry-recognized quality thresholds	0.838				
QV4	The efficiency of tourism services is high	0.786				
Emotional value			0.885	74.103	0.659	0.885
EV1	A sustained state of psychological fulfillment and physiological comfort	0.742				
EV2	Stimulated a sense of exhilaration	0.807				
EV3	Made me related	0.842				
EV4	Made me feel happy	0.85				
Price value			0.867	71.3	0.621	0.873
PV1	Delivered equitable returns on monetary investment relative	0.732				
PV2	Optimal alignment with market expectations for comparable service tiers	0.755				
PV3	Good one for the price paid	0.845				
PV4	The pricing structure adheres to affordability thresholds	0.815				
Novelty value			0.845	68.179	0.578	0.845
NV1	Stimulated my sense of exploration and discovery	0.683				
NV2	Addressed my desire for novel cultural encounters	0.805				
NV3	Provided genuine immersion in local traditions	0.792				
NV4	Enabled comprehensive engagement with diverse aspects of the destination’s cultural	0.756				
Tourist satisfaction			0.875	72.354	0.633	0.873
TS1	Generally satisfied	0.742				
TS2	All expectations are fulfilled	0.811				
TS3	Have fun	0.786				
TS4	Time and money spent are satisfied	0.839				
Tourist loyalty			0.84	75.835	0.647	0.845
TL1	Stay here again	0.696				
TL2	Others are recommended to travel here	0.898				
TL3	Share travel updates via social media (WeChat, QQ, etc.)	0.806				

SFL = Standardized Factor Loading; VCC = variance cumulative contribution; α = Cronbach’s alpha.

### 4.3 Discriminant validity

To assess the construct discriminability among the first-order factors associated with cultural distortion risk, we first evaluate the discriminant validity of tourists’ perceived value. The results indicate that the proposed three-factor model exhibits the best data fit. As illustrated in Table 6, the data fit of the three-factor model for cultural distortion risk surpasses that of the two-factor model (∆χ² = 102.499, ∆df = 2, p < 0.0001). Additionally, the fit of the three-factor model is superior to that of the second-order factor model and the single-factor model. This comparative analysis demonstrates that the factors contributing to cultural distortion risk possess strong discriminant validity and effectively represent three distinct constructs [46].

**Table 6:** Results of the perceived value discriminant validity test.

Model	χ2	df	χ2/df	NFI	CFI	RMSEA	Model Comparison	∆χ2	∆df
Three factor model	65.807	51	1.29	0.978	0.995	0.028			
Two factor model Ⅰ	168.306	53	3.176	0.944	0.961	0.112	2 VS 1	102.499***	2
Two factor model Ⅱ	120.654	53	1.937	0.966	0.983	0.05	3 VS 1	54.847***	2
Single factor model	244.054	54	4.52	0.918	0.935	0.096	4 VS 1	178.247***	3

***p < 0.001, Two factor model Ⅰ = F1 + F2, F3; Two factor model Ⅱ = F2 + F3, F1; Single factor model = F1 + F2 + F3; F1: cultural ontology distortion; F2: cultural representation distortion; F3: cultural construction distortion.

Discriminant validity was assessed using the Fornell-Larcker criterion [67]. The square roots of the AVE for all perceived value constructs (ranging from 0.760 to 0.812, Table 7) exceeded the corresponding inter-construct correlations, thus confirming adequate discriminant validity.

**Table 7:** Results of the perceived value discriminant validity test.

Variable	Quality value	Emotional value	Price value	Novelty value
Quality value	**0.780**			
Emotional value	0.708**	**0.812**		
Price value	0.674**	0.715**	**0.788**	
Novelty value	0.524**	0.677**	0.664**	**0.760**

**p < 0.01.

### 4.4 Model fit

**Fig 4:**
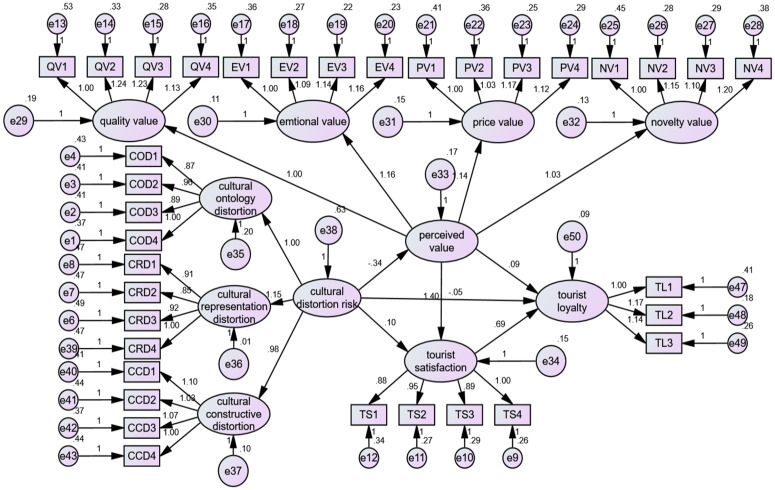
SEM analysis.

SEM was implemented in Amos (Fig 4). The model demonstrated excellent fit to the data: χ²/df = 1.567, RMSEA = 0.039, GFI = 0.964, IFI = 0.964, TLI = 0.961, with all indices meeting established thresholds for good model fit [68].

### 4.5 Hypothesis testing

#### 4.5.1 Direct effects.

The results of the SEM direct path analysis show in Table 8 that the standardized coefficients for cultural distortion risk on perceived value, tourist satisfaction, and tourist loyalty are −0.544 (t = −7.649, p < 0.001) and −0.055 (t = −1.142, p > 0.05), Separately. This finding confirms H1 while rejecting H1a and H1b, suggesting that cultural distortion risk has a direct and substantial impact on perceived value. Nonetheless, its influence on satisfaction and loyalty is not statistically meaningful. Additionally, the standardized coefficients for perceived value on satisfaction and loyalty are 0.909 (t = 9.819, p < 0.001), respectively. Consequently, H2a is supported while H2b is not, indicating that perceived value directly influences satisfaction, but its effect on loyalty is insignificant. Finally, the standardized coefficient for tourist satisfaction on loyalty is 0.804 (t = 7.170, p < 0.001), confirming that H3a is valid and that tourist satisfaction has a strong and direct positive impact on loyalty.

**Table 8:** Results of model direct path relationship test.

Hypotheses	β	T-value	P-value	Supported
H1 CDR → PEV	−0.544	−7.649	***	Yes
H1a CDR → TS	0.102	1.998	0.046	Yes
H1b CDR → TL	−0.055	−1.142	0.254	No
H2a PEV → TS	0.909	9.819	***	Yes
H2b PEV → TL	0.072	0.634	0.526	No
H3a TS → TL	0.804	7.170	***	Yes

CDR = Cultural distortion risk; PEV = Perceived value; TS = Tourist satisfaction; TL = Tourist loyalty.

#### 4.5.2 Indirect effects.

Bootstrap analysis (5,000 samples, 95% CI) was performed in AMOS to test mediation effects [69]. Results indicated no significant direct mediation through perceived value (H2) or satisfaction (H3) between cultural distortion risk and tourist loyalty. However, a significant serial mediation effect was confirmed via the path cultural distortion risk → perceived value → satisfaction → loyalty (β = −0.21, 95% CI [−0.32, −0.11]), supporting H4 (Table 9).

**Table 9:** Results of model indirect path relationship test.

Hypotheses	Effect	β	95% confidence interval		P
			LB	UB	
CDR → TL	Total effcet	−0.409	−0.516	−0.297	0.000
	Direct effect	−0.055	−0.163	0.042	0.276
	CDR → TS → TL	0.082	−0.008	0.192	0.071
	CDR → PEV → TL	−0.039	−0.186	0.118	0.608
	CDR → PEV → TS → TL	−0.397	−0.579	−0.258	0.000
	Total indirect effect	−0.354	−0.474	−0.231	0.000

CDR = Cultural distortion risk; TL = Tourist loyalty; TS = Tourist satisfaction; PV = Perceived value.

## 5 Discussion

### 5.1 H1: Cultural distortion risk → Perceived value (Supported)

Consistent with Leong et al. (2023) [39] and Chen (2023) [42], the data confirm that perceived cultural distortion risk exerts a strong negative influence on perceived value (β = –0.544, p < 0.001). This result reinforces the theoretical expectation that tourists cognitively discount the worth of their heritage experience when they sense that authenticity has been compromised by commercialisation or narrative simplification [29]. The finding aligns with recent cave-heritage studies where physical deterioration and restoration interventions jointly erode epistemic and emotional value [29], thus validating the role of cultural distortion risk as a salient negative stimulus in the SOR framework.

### 5.2 H1a: Cultural distortion risk → Tourist satisfaction (Not Supported)

Contrary to the hypothesised direct effect, cultural distortion risk does not significantly predict tourist satisfaction (β = 0.102, p = 0.046) once perceived value is entered into the model. This null finding can be interpreted through two mechanisms. First, satisfaction formation in heritage settings is predominantly value-driven rather than risk-driven [33]. Tourist appear to translate their perception of distortion into dissatisfaction only after first re-evaluating the overall value of the visit. Second, affective adaptation may be at play—tourists who anticipate some degree of commodification at a mass-tourism site such as Maijishan may lower their affective baseline, thereby muting a direct cultural distortion risk–satisfaction link. These results echo Jebbouri’s (2021) [37] observation that authenticity perceptions lose explanatory power for satisfaction once perceived value is controlled.

### 5.3 H1b: Cultural distortion risk → Tourist loyalty (Not Supported)

The absence of a direct Cultural distortion risk → Tourist loyalty path (β = –0.055, p = 0.254) appears counter-intuitive given the central thesis that distortion erodes loyalty. However, the total effect of cultural distortion risk on tourist loyalty is significantly negative (β = –0.409, p < 0.001) and almost fully mediated by perceived value and tourist satisfaction. This pattern suggests that tourists do not “punish” the site at the behavioural level unless the distortion has already devalued their experience and reduced satisfaction. The finding corroborates Wang’s (2023) meta-analysis [34], which shows that loyalty is rarely affected by isolated negative stimuli but rather by the sequential cognitive-affective appraisal cascade.

### 5.4 H2a: Perceived value → Tourist satisfaction (Supported)

The path coefficient (β = 0.909, p < 0.001) corroborates the dominant paradigm in heritage tourism research that perceived value is the primary antecedent of tourist satisfaction [32,33]. The exceptionally high effect size indicates that, at Maijishan, satisfaction is almost entirely contingent on tourists’ holistic evaluation of functional, emotional, and epistemic benefits. This is consistent with experiential accounts where awe-inspiring Buddhist art and interpretive depth enhance satisfaction only when perceived as “worth the effort” [47].

### 5.5 H2b: Perceived value → Tourist loyalty (Not supported)

The non-significant path (β = 0.072, p = 0.526) deviates from prior studies [34]. A plausible explanation is the near-perfect mediation by satisfaction; once tourist satisfaction is introduced, the residual variance in tourist loyalty is almost completely explained by affective fulfilment rather than by rational value assessment. This suggests that at high-sacrifice, high-involvement heritage sites, emotional closure is a prerequisite for translating value into future behavioural intent.

### 5.6 H3a: Tourist satisfaction → Tourist loyalty (Supported)

The strong positive effect (β = 0.804, p < 0.001) is in line with expectation-confirmation theory and corroborates findings from Liangzhu [61] and red-tourism contexts [15]. It underscores that affective states formed on-site are the most proximal drivers of revisit and recommendation intentions, reaffirming the primacy of emotional experience in heritage loyalty formation [53].

### 5.7 H4: Chain mediation cultural distortion risk → Perceived value → Tourist satisfaction → Tourist loyalty (Supported)

Bootstrapped results confirm the serial mediation (β = –0.397, 95% CI [–0.579, –0.258]), indicating that cultural distortion risk erodes loyalty through the sequential cognitive-affective mechanism stipulated by the SOR paradigm. This full-chain mediation reconciles the seemingly conflicting direct effects: Cultural distortion risk is distal and must first contaminate value perceptions, which then dampen satisfaction and ultimately loyalty. The finding extends Zhu’s (2025) [16] experiential-tourism model to a transcultural heritage context and validates the integration of KAP and SOR frameworks proposed in this study.

## 6 Conclusion

### 6.1 Conclusion

This study establishes that: (1) Cultural Distortion Risk at Maijishan Grottoes does not directly erode Tourist Satisfaction or Loyalty. (2) Instead, its detrimental impact is fully mediated through a sequential cognitive-affective chain: Cultural distortion risk → Perceived Value → Tourist satisfaction → Tourist loyalty(β = −0.397, 95% CI [−0.579, −0.258]). (3) Dimensional analysis reveals that cultural representation distortion (β = −0.38) and cultural ontology distortion (β = −0.32; Cohen’s d = 0.62, RW = 41.3%) exert the strongest effects on perceived value degradation, collectively accounting for 77% of recoverable value loss. (4) Perceived value’s erosion is primarily driven by declines in emotional value (β = 0.45) and educational value (β = 0.41), with tourist satisfaction mediating 89.7% of perceived value’s total effect on tourist loyalty (β = 0.804, p < 0.001).

### 6.2 Implications

#### 6.2.1 Theoretical implications.

Theoretically, this research advances heritage-tourism theory by: (1) Validating a reliable second-order cultural distortion risk scale (α = 0.955) that encompasses the dimensions of ontology, representation, and construction. The cultural distortion risk at heritage sites model, developed by Zhang et al (2024) [29], was validated. The findings indicate that the perception of cultural distortion risk indirectly diminishes loyalty through a chain mediation effect, thereby providing substantial support for the cross-scenario applicability of the CDRHS model. This validation not only reinforces the theoretical significance of cultural risk in tourism research but also broadens the model’s applicability, elucidating the multi-stage mechanism through which cultural distortion risk influences tourist behavior. This accomplishment offers a replicable analytical framework for future research. (2) This study demonstrates a cognition-affect-behavior cascade via an integrated SOR/KAP model and thus challenges the linear-risk-aversion assumption. Perceived value and satisfaction serve as chain mediators between the risk of cultural distortion and loyalty. This conclusion synthesizes the fundamental principles of the KAP model and ROS theory. Specifically, the risk of cultural distortion diminishes tourists’ rational evaluation of heritage value (perceived value), which in turn weakens their emotional experiences (satisfaction) and ultimately impacts their behavioral loyalty. This pathway illustrates the progressive transformation of risk perception from cognitive to emotional dimensions, addressing the limitations of the traditional single ‘risk-behavior’ mediating pathway and highlighting the synergistic role of multidimensional psychological mechanisms. This finding exemplifies interdisciplinary theoretical integration and proposes a dual-layer intervention strategy of ‘value reshaping-emotional connection’ aimed at enhancing tourist experiences at heritage sites. (3) Quantifying hierarchical mediation effects and confirming that perceived value and trust in services serve as serial buffers against cultural dissonance risk. The integrated model of “Cultural Distortion Risk-Perceived Value-Satisfaction-Loyalty” constructed in this research supply a systematic theoretical framework for the management practices of Silk Road cultural heritage sites. The model not only clarifies the negative impact mechanism of cultural risk on tourist loyalty but also identifies key intervention points through a chain mediation path. This framework addresses the deficiency of “emphasizing protection over experience” in existing cultural heritage management theories. The theoretical framework constructed in this study has significant cross-regional adaptability, providing a reusable scientific paradigm for the protection and development of similar cultural heritage sites. Its innovative methodology not only expands the theoretical boundaries of heritage tourism research but also offers a dual guidance system for management decision-making and value transformation mechanism design in the practical field.

#### 6.2.2 Management implications.

For management, evidence-based prioritization is critical: (1) Target high-impact cultural distortion risk dimensions by restoring spiritual depth through monastic co-created narratives (ontology, RW 41.3%) and enforcing authenticity certification for performances and souvenirs (representation, β = −0.38). The “Grotto Genesis” narrative track explains how cave temples served as a reflective corridor through which Buddhism was indigenized in China. Consequently, a “Maijishan Authentication” system has been established, encompassing every physical artifact to safeguard cultural authenticity. Deep monastic engagement imbues the heritage with a living spirit, while the rigorous authentication protocol ensures the purity of its transmission channels. These two dimensions are mutually reinforcing and effectively mitigate the risk of “cultural distortion” inherent in dissemination and commodification. This dual mechanism not only guarantees the faithful communication of the grottoes’ millennia-old artistic charisma but also allows their embedded wisdom and ethos of equanimity to regain contemporary relevance. (2) Amplify dominant perceived value drivers by designing immersive storytelling routes (emotional value, β = 0.45) and deploying virtual reality reconstructions of historical states (educational value, β = 0.41). Therefore, the Maijishan Grottoes should prioritize the implementation of measures to safeguard cultural authenticity, including limiting the daily number of visitors, restoring weathered murals, and prohibiting inappropriate commercial developments, such as shops or entertainment facilities that are not aligned with the grotto’s cultural heritage. Drawing inspiration from the ‘digital diversion’ model employed at the Mogao Grottoes, virtual reality technology can be leveraged to showcase details of specific caves, thereby mitigating the physical damage to artifacts caused by in-person visits. This approach not only reduces the risk of cultural distortion but also enhances visitors’ rational understanding of the historical and artistic significance of the grottoes through authentic presentations, thereby increasing their perceived value. Additionally, an intelligent cultural interpretation system should be established, and smart tour guide tools should be developed to ensure that visitors accurately grasp the cultural essence of the grottoes, thus preventing cultural misinterpretation stemming from information bias. (3) Monitor satisfaction thresholds by implementing real-time satisfaction tracking (e.g., mobile apps) and triggering interventions if tourist satisfaction falls below 4.0 out of 5.0. By optimizing the chain experience, the negative impact of cultural distortion risks can be transformed into opportunities that enhance visitor loyalty. Strengthening the cultural promotion of the scenic area will also elevate the cultural perception among potential visitors. By effectively promoting the cultural content of the Maijishan Grottoes, the cultural value is intricately embedded into the cognition of potential tourists. This approach not only mitigates the negative impact of cultural distortion risks on perceived value, but also fosters emotional identification in advance. This creates prerequisites for enhanced satisfaction during on-site visits and long-term loyalty behaviors, ultimately promoting the healthy, stable, and sustainable development of cultural heritage tourism.

### 6.3 Limitations

Three constraints warrant acknowledgment: (1) The cross-sectional nature of the data limits the ability to draw causal inferences beyond the structural model. (2) The sampling bias towards educated, high-income visitors reduced the generalizability of findings to mass-market segments. (3) The spiritual context of the Buddhist grottoes may mitigate dissatisfaction related to cultural distortion ratings, thereby constraining the extrapolation of effect sizes to secular sites (e.g., ruins, cultural landscapes).

### 6.4 Future research

To address the nonsignificant direct effects of cultural distortion risk on tourist satisfaction and tourist loyalty and to enhance generalizability, future research should focus on the following three aspects. (1) Investigate cultural and situational attenuators: Explore cultural moderators (e.g., collectivism, heritage salience) and contextual factors (e.g., crowd density) that may explain the rejection of hypotheses H1a and H1b. (2) Test authenticity interventions: Utilize longitudinal and experimental designs (e.g., augmented reality interpretation, capacity-controlled tours) to quantify the efficacy of cultural distortion risk mitigation. (3) Enable cross-context validation: Replicate the model across at least three Silk Road sites using standardized distortion metrics (e.g., UNESCO 2023) and incorporate cross-cultural comparisons among tourists.

This evidence hierarchy highlights that tourists penalize heritage sites only when distortion first diminishes cognitive appraisal and subsequently dampens affective fulfillment. Therefore, proactive preservation of high-leverage value dimensions represents the most effective strategy for sustaining tourist loyalty.

## Supporting information

S1 Data Questionnaire data. https://doi.org/10.6084/m9.figshare.30264874.
(XLSX)

S1 Fig Structural equation modeling supplementary figure. (PDF)

S1 Table Structural equation modeling supplementary table. (DOCX)

S1 File Questionnaire. (DOCX)

S2 File Survey questionnaire informed consent form. (PDF)

## References

[1] Extended 45th session of the World Heritage Committee. 2023. https://whc.unesco.org/archive/2023/whc23-45com-19-en.pdf

[2] International Council on Monuments and Sites (ICOMOS). International charter for cultural heritage tourism: Reinforcing cultural heritage protection and community resilience through responsible and sustainable tourism management. ICOMOS. http://www.icomoschina.org.cn/content/details48_9834.html

[3] Zhang S, Liang J, Su X, Chen Y, Wei Q. Research on global cultural heritage tourism based on bibliometric analysis. Herit Sci. 2023;11(1). doi: 10.1186/s40494-023-00981-w

[4] Werner C. The New Silk Road: Mediators and Tourism Development in Central Asia. Ethnology. 2003;42(2):141. doi: 10.2307/3773779

[5] Manhas PS, Kour P, Bhagata A. Silk Route in the Light of Circuit Tourism: An Avenue of Tourism Internationalization. Procedia - Social and Behavioral Sciences. 2014;144:143–50. doi: 10.1016/j.sbspro.2014.07.283

[6] Brown MS, O’Brien D. The silk roads and shared heritage in Europe: Beyond ‘China to Rome’. China Information. 2024. doi: 10.1177/0920203x241281989

[7] Yu J, Safarov B, Yi L, Buzrukova M, Janzakov B. The Adaptive Evolution of Cultural Ecosystems along the Silk Road and Cultural Tourism Heritage: A Case Study of 22 Cultural Sites on the Chinese Section of the Silk Road World Heritage. Sustainability. 2023;15(3):2465. doi: 10.3390/su15032465

[8] Zhu Z, Qin Y, Guo Z, Cai S, Lin P, Wang X, et al. Shedding new light on lacquering crafts from the Northern Wei Dynasty (386–534 CE) by revisiting the lacquer screen from Sima Jinlong’s Tomb. Journal of Cultural Heritage. 2025;71:309–19. doi: 10.1016/j.culher.2024.12.002

[9] Zhang K, González del Valle-Brena A, Ramos Riera I, Zhao JL. (2024) Ancient routes, new gateways: a systematic literature review of China’s cultural route heritage. Journal of Cultural Heritage Management and Sustainable Development 14 (2): 266–81. doi: 10.1108/JCHMSD-06-2021-0114

[10] Corsale A. Jewish Heritage Tourism in Krakow. Authenticity and Commodification Issues. Tourism and Hospitality. 2021;2(1):140–52. doi: 10.3390/tourhosp2010008

[11] Zhang T, Yin P, Peng Y. Effect of Commercialization on Tourists’ Perceived Authenticity and Satisfaction in the Cultural Heritage Tourism Context: Case Study of Langzhong Ancient City. Sustainability. 2021;13(12):6847. doi: 10.3390/su13126847

[12] Zhang Y. Analysis on the Spread Law of Dunhuang Dance Culture. JHASS. 2023;7(7):1275–8. doi: 10.26855/jhass.2023.07.005

[13] Mathew E. Globalization and Local Flavours: The Impact of Modern Food Production on Traditional Cuisine and Culinary Heritage Preservation. IJMRP. 2024;2(7):61–74. doi: 10.61877/ijmrp.v2i7.170

[14] UNESCO. Convention Concerning the Protection of the World Cultural and Natural Heritage. Dictionary of Geotourism. Springer Singapore. 2019. 97–8. doi: 10.1007/978-981-13-2538-0_398

[15] Dai Q, Chen J, Zheng Y. Assessing the impact of community-based homestay experiences on tourist loyalty in sustainable rural tourism development. Sci Rep. 2025;15(1):122. doi: 10.1038/s41598-024-84075-y 39747506 PMC11696156

[16] Zhu N, Xu H, Zhang X, Lu Y. Research on the factors influencing tourist loyalty to outdoor music festivals: an application of stimulus-organism-response paradigm. Front Psychol. 2025;16:1553211. doi: 10.3389/fpsyg.2025.1553211 40475337 PMC12139457

[17] Liu S, Wang X, Wang L, Pang Z. Influence of Non-Standard Tourist Accommodation’s Environmental Stimuli on Customer Loyalty: The Mediating Effect of Emotional Experience and the Moderating Effect of Personality Traits. Int J Environ Res Public Health. 2022;19(15):9671. doi: 10.3390/ijerph19159671 35955025 PMC9367805

[18] Lee S, Phau I, Hughes M, Li YF, Quintal V. Heritage Tourism in Singapore Chinatown: A Perceived Value Approach to Authenticity and Satisfaction. Journal of Travel & Tourism Marketing. 2016;33(7):981–98. doi: 10.1080/10548408.2015.1075459

[19] Poria Y, Butler R, Airey D. The core of heritage tourism. Annals of Tourism Research. 2003;30(1):238–54. doi: 10.1016/s0160-7383(02)00064-6

[20] Lan T, Zheng Z, Tian D, Zhang R, Law R, Zhang M. Resident-Tourist Value Co-Creation in the Intangible Cultural Heritage Tourism Context: The Role of Residents’ Perception of Tourism Development and Emotional Solidarity. Sustainability. 2021;13(3):1369. doi: 10.3390/su13031369

[21] Antonio Parrilla González J, Ortega Alonso D. Sustainable Development Goals in the Andalusian olive oil cooperative sector: Heritage, innovation, gender perspective and sustainability. New Medit. 2022;21(02). doi: 10.30682/nm2202c

[22] Fu XY, Li Y, Sun ZJ, Du J, Wang FP, Xu YQ. Digital color restoration of soot covered murals in the Mogao Grottoes at Dunhuang. Journal of Dunhuang Academy. 2021;(01):137–47. doi: 10.13584/j.cnki.issn1000-4106.2021.01.019

[23] Putra H. Indonesia Towards Ratification of the 2001 Convention on Underwater Cultural Heritage Protection: Challenges and Opportunities. J Mari Arch. 2025;20(2):331–49. doi: 10.1007/s11457-025-09444-8

[24] Juanis B, Saleh Y, Ghazali MKA, Mahat H, Hashim M, Nayan N, et al. Knowledge, Attitudes and Practices of Youths Towards the Intangible Cultural Heritage Elements of Dusun Ethnic in Malaysian Environment. IOP Conf Ser: Earth Environ Sci. 2022;975(1):012008. doi: 10.1088/1755-1315/975/1/012008

[25] Hu J, Noor SM. Knowledge, attitudes and practices of intangible cultural heritage among youth in Sichuan, China: a cross-sectional study. Journal of Cultural Heritage Management and Sustainable Development. 2024. doi: 10.1108/JCHMSD-10-2023-0174

[26] Huang R, Bu H-M. Destination Attributes of Memorable Chinese Rural Tourism Experiences: Impact on Positive Arousal, Memory and Behavioral Intention. Psychol Res Behav Manag. 2022;15:3639–61. doi: 10.2147/PRBM.S387241 36540859 PMC9760039

[27] Hernández-Rojas RD, Huete Alcocer N. The role of traditional restaurants in tourist destination loyalty. PLoS One. 2021;16(6):e0253088. doi: 10.1371/journal.pone.0253088 34138912 PMC8211172

[28] Williamson J, Hassanli N. It’s all in the recipe: How to increase domestic leisure tourists’ experiential loyalty to local food. Tour Manag Perspect. 2020;36:100745. doi: 10.1016/j.tmp.2020.100745 32953431 PMC7491424

[29] Zhang S-N, Ruan W-Q, Li Y-Q, Huang H. Local cultural distortion risk at tourist destinations: connotation deconstruction and theoretical construction. Current Issues in Tourism. 2023;27(2):251–67. doi: 10.1080/13683500.2023.2178393

[30] Wahid SS, Ottman K, Hudhud R, Gautam K, Fisher HL, Kieling C, et al. Identifying risk factors and detection strategies for adolescent depression in diverse global settings: A Delphi consensus study. J Affect Disord. 2021;279:66–74. doi: 10.1016/j.jad.2020.09.098 33039776 PMC7758738

[31] Ameen S, Faye A. Role of media - social, electronic, and print media - in mental health and wellbeing. Indian J Psychiatry. 2024;66(Suppl 2):S403–13. doi: 10.4103/indianjpsychiatry.indianjpsychiatry_611_23 38445281 PMC10911332

[32] Wang H, Yang Y, He W. Does Value Lead to Loyalty? Exploring the Important Role of the Tourist-Destination Relationship. Behav Sci (Basel). 2022;12(5):136. doi: 10.3390/bs12050136 35621433 PMC9138082

[33] Qiu N, Li H, Pan C, Wu J, Guo J. The study on the relationship between perceived value, satisfaction, and tourist loyalty at industrial heritage sites. Heliyon. 2024;10(17):e37184. doi: 10.1016/j.heliyon.2024.e37184 39286155 PMC11403092

[34] Wang L, Li X. The five influencing factors of tourist loyalty: A meta-analysis. PLoS One. 2023;18(4):e0283963. doi: 10.1371/journal.pone.0283963 37040349 PMC10089331

[35] Xiong S, Zhang T. Enhancing tourist loyalty through location-based service apps: Exploring the roles of digital literacy, perceived ease of use, perceived autonomy, virtual-content congruency, and tourist engagement. PLoS One. 2024;19(1):e0294244. doi: 10.1371/journal.pone.0294244 38295124 PMC10830003

[36] Yang S, Isa SM, Yao Y, Xia J, Liu D. Cognitive image, affective image, cultural dimensions, and conative image: A new conceptual framework. Front Psychol. 2022;13:935814. doi: 10.3389/fpsyg.2022.935814 35983200 PMC9378820

[37] Jebbouri A, Zhang H, Wang L, Bouchiba N. Exploring the Relationship of Image Formation on Tourist Satisfaction and Loyalty: Evidence From China. Front Psychol. 2021;12:748534. doi: 10.3389/fpsyg.2021.748534 34887804 PMC8649662

[38] Cao EY, Chong KM, Pan L, Ning L, Pan FD, Li KK. Oh I remember the beauty and aesthetics of guilin!: Exploring the implications of memorability on tourist loyalty through a two-wave panel data. Heliyon. 2024;10(1):e23365. doi: 10.1016/j.heliyon.2023.e23365 38169803 PMC10758774

[39] Leong AMW, Yeh S-S, Zhou Y, Hung C-W, Huan T-C. Exploring the influence of historical storytelling on cultural heritage tourists’ value co-creation using tour guide interaction and authentic place as mediators. Tourism Management Perspectives. 2023;50:101198. doi: 10.1016/j.tmp.2023.101198

[40] Nursanty E, Rusmiatmoko D, Muhammad Fahd Diyar Husni. From Heritage to Identity: The Role of City Authenticity in Shaping Local Community Identity and Cultural Preservation. AJAHE. 2023;1(2):131–50. doi: 10.59810/archimane.v1i2.17

[41] Liu Z, Zhang M, Osmani M. Building Information Modelling (BIM) Driven Sustainable Cultural Heritage Tourism. Buildings. 2023;13(8):1925. doi: 10.3390/buildings13081925

[42] Chen D. How Visitors Perceive Heritage Value—A Quantitative Study on Visitors’ Perceived Value and Satisfaction of Architectural Heritage through SEM. Sustainability. 2023;15(11):9002. doi: 10.3390/su15119002

[43] Dipierro AR, Rella A. What Lies Behind Perceptions of Corruption? A Cultural Approach. Soc Indic Res. 2024;172(2):371–91. doi: 10.1007/s11205-023-03294-4

[44] Yasir N, Mahmood N, Mehmood HS, Babar M, Irfan M, Liren A. Impact of Environmental, Social Values and the Consideration of Future Consequences for the Development of a Sustainable Entrepreneurial Intention. Sustainability. 2021;13(5):2648. doi: 10.3390/su13052648

[45] Wu Z, Liu Y. Exploring country differences in the adoption of mobile payment service: the surprising robustness of the UTAUT2 mode. International Journal of Bank Marketing 2022:41(2): 237–68. doi: 10.1108/ijbm-02-2022-0052

[46] Chen Q, Xu S, Liu R, Jiang Q. Exploring the Discrepancy between Projected and Perceived Destination Images: A Cross-Cultural and Sustainable Analysis Using LDA Modeling. Sustainability. 2023;15(12):9296. doi: 10.3390/su15129296

[47] Feng Y, Pan J. How Does Prior Distribution Affect Model Fit Indices of Bayesian Structural Equation Model?. Fudan J Hum Soc Sci. 2024;18(1):137–73. doi: 10.1007/s40647-024-00416-1

[48] Schumann M, Dennis A, Leduc J-M, Peters H. Translating cross-language qualitative data in health professions education research: Is there an iceberg below the waterline?. Med Educ. 2024;59(6):589–95. doi: 10.1111/medu.15563 39484704 PMC12070362

[49] Piggin J, Batten J, Parry K, Anderson E, White AJ. Compulsory collisions and corporate interests in school rugby: challenging distortions in the framing of childhood injury. Inj Prev. 2022;29(1):79–84. doi: 10.1136/ip-2022-044775 36376056

[50] Kocsis J. ’¡Eso no se dice’!: Exploring the value of communication distortions in participatory planning. Plan Theory. 2023;22(3):270–91. doi: 10.1177/14730952221124824 37539367 PMC10394399

[51] Maags C. Disseminating the policy narrative of ‘Heritage under threat’ in China. International Journal of Cultural Policy. 2018;26(3):273–90. doi: 10.1080/10286632.2018.1500559

[52] Li J, Xue E. Shaping the Educational Value in China: Ideas and Practices. Exploring Education Policy in a Globalized World: Concepts, Contexts, and Practices. Springer Singapore. 2020. 29–37. doi: 10.1007/978-981-15-7745-1_2

[53] Jimber Del Río JA, Hernández-Rojas RD, Vergara-Romero A, Dancausa Millán MGD. Loyalty in Heritage Tourism: The Case of Córdoba and Its Four World Heritage Sites. Int J Environ Res Public Health. 2020;17(23):8950. doi: 10.3390/ijerph17238950 33271956 PMC7731023

[54] Calado F, Vernon M, Nuyens F, Alexandre J, Griffiths MD. How Does Religiosity Influence Gambling? A Cross-Cultural Study Between Portuguese and English Youth. J Gambl Stud. 2023;40(2):1005–19. doi: 10.1007/s10899-023-10269-0 38070070 PMC11272725

[55] Mohamed BAA, Idrees MHD. Body image dissatisfaction and its relation to body mass index among female medical students in Sudan: across-sectional study 2020-2021. BMC Womens Health. 2023;23(1):593. doi: 10.1186/s12905-023-02748-8 37950174 PMC10638698

[56] Salganik MJ, Watts DJ. Leading the Herd Astray: An Experimental Study of Self-Fulfilling Prophecies in an Artificial Cultural Market. Soc Psychol Q. 2008;74(4):338. doi: 10.1177/019027250807100404 24078078 PMC3785310

[57] Regalado-Pezúa O, Carvache-Franco M, Carvache-Franco O, Carvache-Franco W. Perceived value and its relationship to satisfaction and loyalty in cultural coastal destinations: A study in Huanchaco, Peru. PLoS One. 2023;18(8):e0286923. doi: 10.1371/journal.pone.0286923 37527247 PMC10393132

[58] Zhang H, Jiang J, Zhu JJ. The perceived value of local knowledge tourism: dimension identification and scale development. Front Psychol. 2023;14:1170651. doi: 10.3389/fpsyg.2023.1170651 37637924 PMC10447968

[59] Oliver RL. A Cognitive Model of the Antecedents and Consequences of Satisfaction Decisions. Journal of Marketing Research. 1980;17(4):460–9. doi: 10.1177/002224378001700405

[60] Shukla A, Mishra A, Dwivedi YK. Expectation confirmation theory: A review. In: Papagiannidis S. TheoryHub Book. 2025.

[61] Fu Y, Luo JM. An empirical study on cultural identity measurement and its influence mechanism among heritage tourists. Front Psychol. 2023;13:1032672. doi: 10.3389/fpsyg.2022.1032672 36743645 PMC9895845

[62] Cui H, Jin B. Research on promoting ethnic communication, exchange and integration through tourism in Tianshui, Gansu Province. Journal of Qinghai University for Nationalities (Social Sciences Edition). 2025;51(01):53–60.

[63] Williams P, Soutar GN. Value, satisfaction and behavioral intentions in an adventure tourism context. Annals of Tourism Research. 2009;36(3):413–38. doi: 10.1016/j.annals.2009.02.002

[64] Lu L, Chi CG, Liu Y. Authenticity, involvement, and image: Evaluating tourist experiences at historic districts. Tourism Management. 2015;50:85–96. doi: 10.1016/j.tourman.2015.01.026

[65] Kolar T, Zabkar V. A consumer-based model of authenticity: An oxymoron or the foundation of cultural heritage marketing?. Tourism Management. 2010;31(5):652–64. doi: 10.1016/j.tourman.2009.07.010

[66] Yi X, Lin VS, Jin W, Luo Q. The Authenticity of Heritage Sites, Tourists’ Quest for Existential Authenticity, and Destination Loyalty. Journal of Travel Research. 2017;56(8):1032–48. doi: 10.1177/0047287516675061

[67] Fornell C, Larcker DF. Structural Equation Models with Unobservable Variables and Measurement Error: Algebra and Statistics. Journal of Marketing Research. 1981;18(3):382. doi: 10.2307/3150980

[68] Wei ZR. Application of Structural Equation Model and AMOS Software. AMM. 2014;687–691:1577–9. doi: 10.4028/www.scientific.net/amm.687-691.1577

[69] Preacher KJ, Hayes AF. Asymptotic and resampling strategies for assessing and comparing indirect effects in multiple mediator models. Behav Res Methods. 2008;40(3):879–91. doi: 10.3758/brm.40.3.879 18697684

