# Supplementary material for: Cultural distortion risk and tourist loyalty at silk road heritage: The mediating roles of perceived value and satisfaction
Source: PLoS One. 2025 Nov 5;20(11):e0335476. doi: 10.1371/journal.pone.0335476 (PMC12588480; doi:10.1371/journal.pone.0335476)
Supplement: S1 Fig — (PDF) [file pone.0335476.s002.pdf]

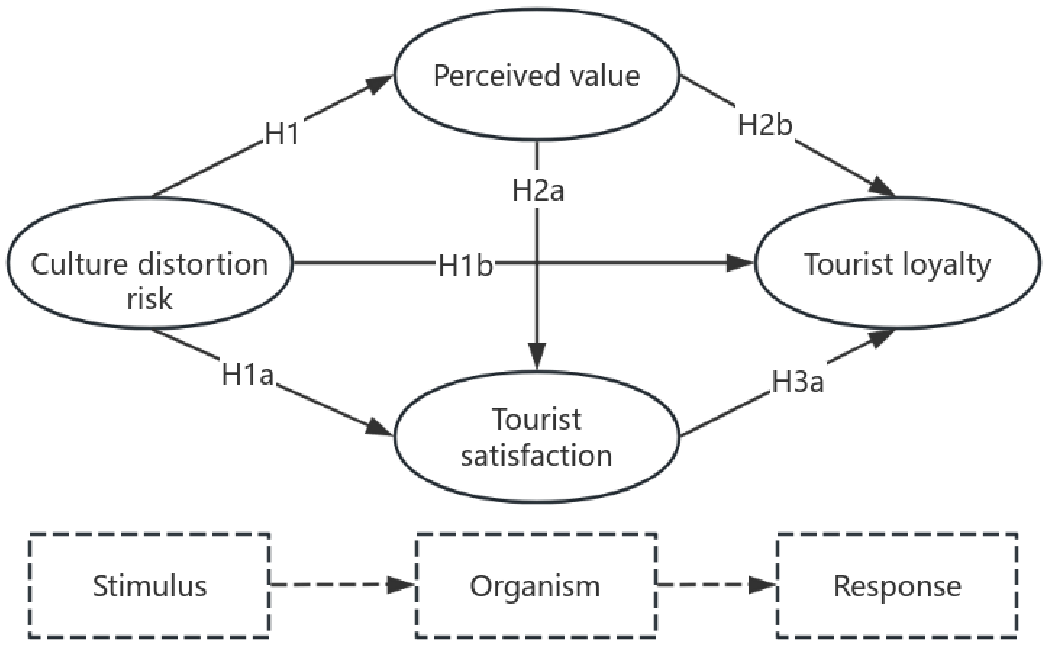

Fig 1. **Research model.**

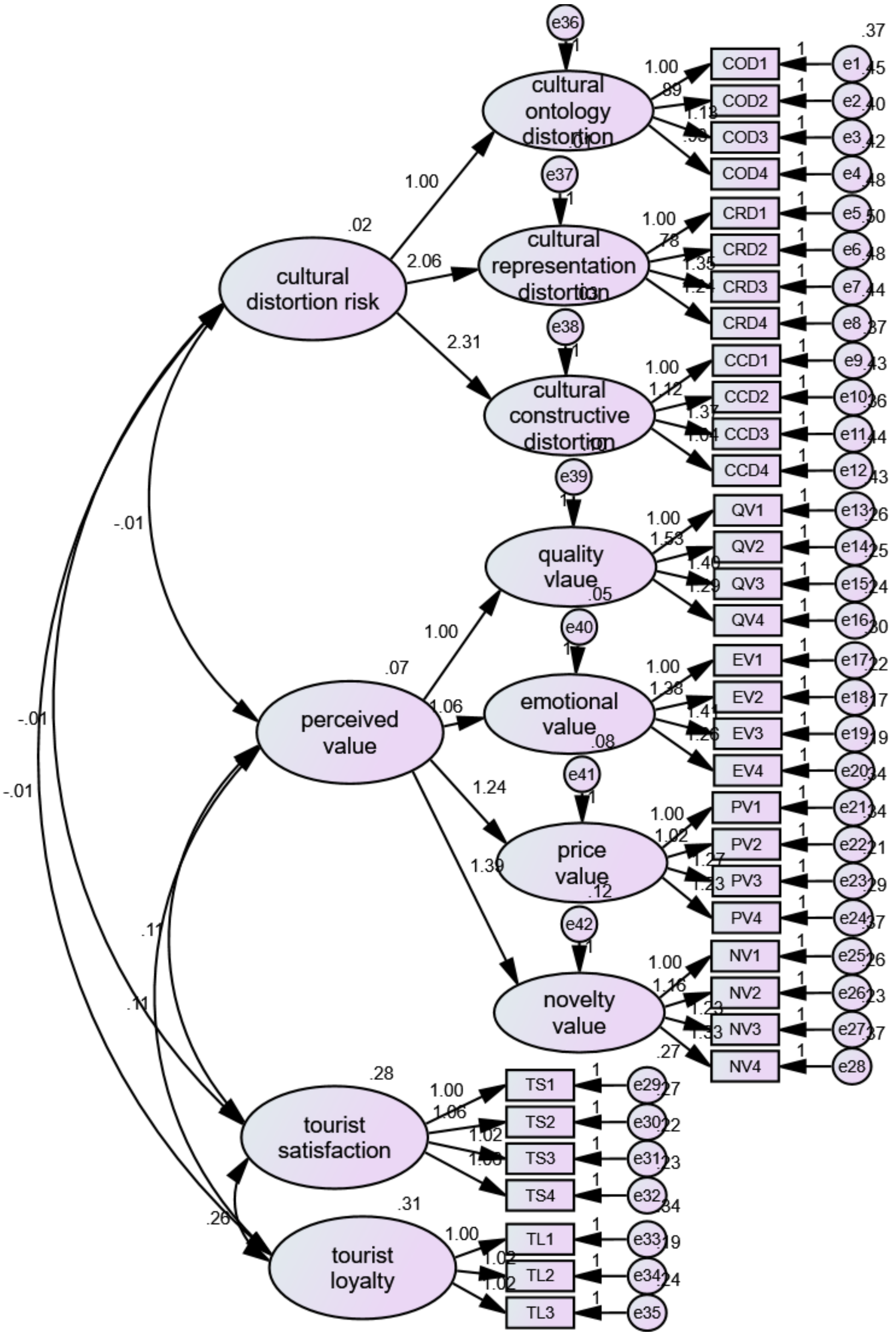

Fig 2. Four factor model analysis.

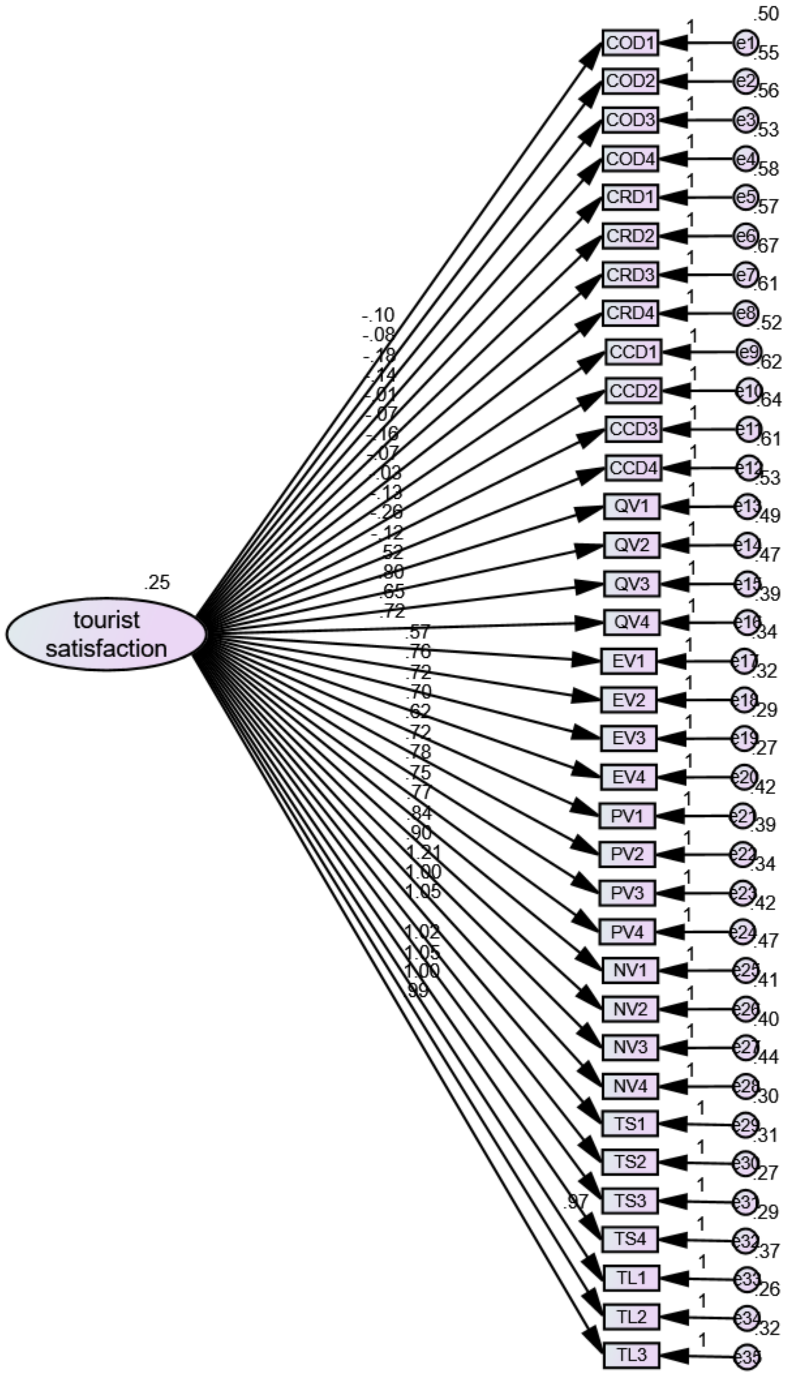

Fig 3. Single factor model analysis.

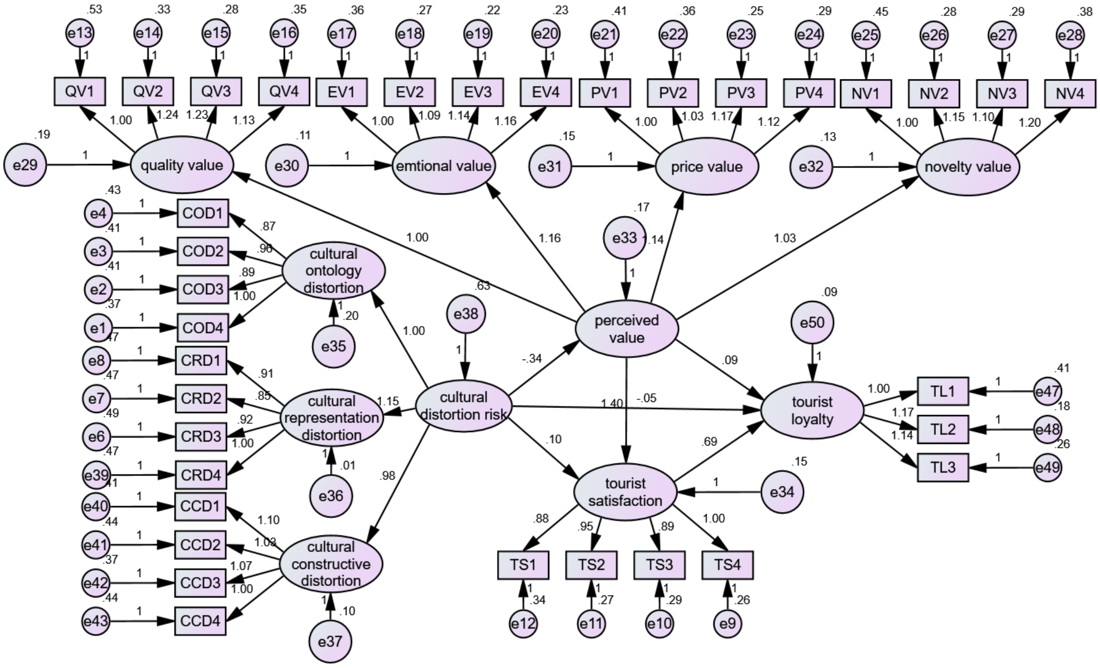

Fig 4. SEM analysis.
