# Supplementary material for: Cultural distortion risk and tourist loyalty at silk road heritage: The mediating roles of perceived value and satisfaction
Source: PLoS One. 2025 Nov 5;20(11):e0335476. doi: 10.1371/journal.pone.0335476 (PMC12588480; doi:10.1371/journal.pone.0335476)
Supplement: S1 Table — (DOCX) [file pone.0335476.s003.docx]

Table1. Measurement items and sources of the questionnaire

| **Variable** | **Dimension** | **Item** | **Source** |
| --- | --- | --- | --- |
| Cultural distortion risk | Cultural  ontology  distortion | Address the challenge of identifying cultural uniqueness | Zhang S. N., et al. (2024) |
|  |  | The difficulty of gaining cultural knowledge |  |
|  |  | The blurred cultural perception |  |
|  |  | The weak cultural impression |  |
|  | Cultural representation distortion | The presentation doesn’t match the original historical style |  |
|  |  | The disconnect between the presentation and the essence of local culture |  |
|  |  | The lack of cultural presentation |  |
|  |  | Cultural products may be cookie−cutter |  |
|  | Cultural  constructive distortion | The original culture will deteriorate |  |
|  |  | The customs and habits will be lost |  |
|  |  | The cultural heritage will be lost |  |
|  |  | Be deviating from the core local culture |  |
| Perceived value | Quality vlaue | Maintained uniform quality standards across multiple iterations | Williams P and Soutar G N. (2009) [65] |
|  |  | The scenic area is well done |  |
|  |  | Met or exceeded industry-recognized quality thresholds |  |
|  |  | The efficiency of tourism services is high |  |
|  | Emotional value | A sustained state of psychological fulfillment and physiological comfort |  |
|  |  | Stimulated a sense of exhilaration |  |
|  |  | Made me related |  |
|  |  | Made me feel happy |  |
|  | Price value | Delivered equitable returns on monetary investment relative |  |
|  |  | Optimal alignment with market expectations for comparable service tiers |  |
|  |  | Good one for the price paid |  |
|  |  | The pricing structure adheres to affordability thresholds |  |
|  | Novelty value | Stimulated my sense of exploration and discovery |  |
|  |  | Addressed my desire for novel cultural encounters |  |
|  |  | Provided genuine immersion in local traditions |  |
|  |  | Enabled comprehensive engagement with diverse aspects of the destination's cultural |  |
| Tourist satisfaction |  | Generally satisfied | Lee et al（2016）[18]；Lu et al（2015）[66] |
|  |  | All expectations are fulfilled |  |
|  |  | Have fun |  |
|  |  | Time and money spent are satisfied |  |
| Tourist loyalty |  | Stay here again | Kolar and Zabkar（2010）[67]；Yi et al（2017）[68] |
|  |  | Others are recommended to travel here |  |
|  |  | Share travel updates via social media (WeChat, QQ, etc.) |  |

Table 2. Measures to mitigate biases in the process of questionnaire data collection

| **Bias Type** | **Mitigation Measure** | **Implementation** |
| --- | --- | --- |
| **Selection Bias** | Stratified time-slot sampling | Offline surveys conducted across 8 time slots (weekdays/weekends, AM/PM) |
| **Non-response Bias** | Incentive equivalence | All participants entered equal lottery for 1×¥5 gift cards regardless of method |
| **Social Desirability** | Anonymity assurance | No personal identifiers collected; cover letter emphasized academic-only use |
| **Measurement Bias** | Blind data entry | Undergraduates performed data entry without access to research hypotheses |
| **Instrument Bias** | Back-translation validation | Bilingual team translated survey into English/Chinese; verified via pilot test (n=30) |

Note(s): Invalid questionnaire criteria: Completion time < 50% median duration (offline: <5 min; online: <3 min), Straight-lining patterns (≥80% identical responses), Contradictory answers to control items (e.g., *"I did not visit MGSA"* vs. site experience questions).

Table 3. Results Common method bias text

| **Model** | **χ2** | **df** | **χ2/df** | **NFI** | **GFI** | **AGFI** | **CFI** | **RMSEA** |
| --- | --- | --- | --- | --- | --- | --- | --- | --- |
| Four factor model | 801.769 | 547 | 1.466 | 0.763 | 0.874 | 0.824 | 0.909 | 0.043 |
| Single factor model | 1705.521 | 520 | 3.046 | 0.496 | 0.668 | 0.626 | 0.589 | 0.091 |

Note(s): four factor model=CFA analysis; single factor model=tourist satisfaction.

Table 4. Demographic variables descriptive statistics

| **Variable** | **Category** | **Frequency** | **Rate（%）** |
| --- | --- | --- | --- |
| Gender | male | 159 | 41.73 |
|  | female | 222 | 58.36 |
| age | 18-29 | 129 | 33.86 |
|  | 30-39 | 110 | 28.87 |
|  | 40-49 | 92 | 24.15 |
|  | 50 and above | 50 | 13.12 |
| Education | Senior middle school and below | 21 | 5.51 |
|  | Junior High | 69 | 18.11 |
|  | Senior High | 85 | 22.31 |
|  | University | 162 | 42.52 |
|  | Master or Doctor | 44 | 11.55 |
| Monthly income(yuan) | 3000 and below | 90 | 23.62 |
|  | 3001-5000 | 91 | 23.88 |
|  | 5001-10000 | 143 | 37.53 |
|  | 10001-20000 | 46 | 12.07 |
|  | 200001 and above | 11 | 2.89 |
| Occupation | Professional | 38 | 9.97 |
|  | Company employee | 56 | 14.70 |
|  | Manufacturer/Technician | 63 | 16.54 |
|  | Service employee | 34 | 8.92 |
|  | Independent businessman | 25 | 6.56 |
|  | Government official/Teacher | 41 | 10.76 |
|  | Student | 59 | 15.49 |
|  | Other | 65 | 17.06 |

Table 5. Results of confirmatory factor analysis.

| **Item codes** | **Questionnaire items** | **SFL** | **α** | **VCC/%** | **AVE** | **CR** |
| --- | --- | --- | --- | --- | --- | --- |
| Cultural distortion risk |  |  |  |  |  | 0.951 |
| Cultural ontology distortion |  |  | 0.874 | 72.648 | 0.612 | 0.863 |
| COD1 | Address the challenge of identifying cultural uniqueness | 0.766 |  |  |  |  |
| COD2 | The difficulty of gaining cultural knowledge | 0.804 |  |  |  |  |
| COD3 | The blurred cultural perception | 0.784 |  |  |  |  |
| COD4 | The weak cultural impression | 0.775 |  |  |  |  |
| Cultural representation distortion |  |  | 0.858 | 70.133 | 0.624 | 0.869 |
| CRD1 | The presentation doesn’t match the original historical style | 0.753 |  |  |  |  |
| CRD2 | The disconnect between the presentation and the essence of local culture | 0.772 |  |  |  |  |
| CRD3 | The lack of cultural presentation | 0.831 |  |  |  |  |
| CRD4 | Cultural products may be cookie−cutter | 0.803 |  |  |  |  |
| Cultural constructive distortion |  |  | 0.88 | 73.508 | 0.648 | 0.88 |
| CCD1 | The original culture will deteriorate | 0.82 |  |  |  |  |
| CCD2 | The customs and habits will be lost | 0.791 |  |  |  |  |
| CCD3 | The cultural heritage will be lost | 0.827 |  |  |  |  |
| CCD4 | Be deviating from the core local culture | 0.781 |  |  |  |  |
| Perceived value |  |  |  |  |  | 0.888 |
| Quality value |  |  | 0.858 | 70.045 | 0.608 | 0.86 |
| QV1 | Maintained uniform quality standards across multiple iterations | 0.675 |  |  |  |  |
| QV2 | The scenic area is well done | 0.81 |  |  |  |  |
| QV3 | Met or exceeded industry-recognized quality thresholds | 0.838 |  |  |  |  |
| QV4 | The efficiency of tourism services is high | 0.786 |  |  |  |  |
| Emotional value |  |  | 0.885 | 74.103 | 0.659 | 0.885 |
| EV1 | A sustained state of psychological fulfillment and physiological comfort | 0.742 |  |  |  |  |
| EV2 | Stimulated a sense of exhilaration | 0.807 |  |  |  |  |
| EV3 | Made me related | 0.842 |  |  |  |  |
| EV4 | Made me feel happy | 0.85 |  |  |  |  |
| Price value |  |  | 0.867 | 71.3 | 0.621 | 0.873 |
| PV1 | Delivered equitable returns on monetary investment relative | 0.732 |  |  |  |  |
| PV2 | Optimal alignment with market expectations for comparable service tiers | 0.755 |  |  |  |  |
| PV3 | Good one for the price paid | 0.845 |  |  |  |  |
| PV4 | The pricing structure adheres to affordability thresholds | 0.815 |  |  |  |  |
| Novelty value |  |  | 0.845 | 68.179 | 0.578 | 0.845 |
| NV1 | Stimulated my sense of exploration and discovery | 0.683 |  |  |  |  |
| NV2 | Addressed my desire for novel cultural encounters | 0.805 |  |  |  |  |
| NV3 | Provided genuine immersion in local traditions | 0.792 |  |  |  |  |
| NV4 | Enabled comprehensive engagement with diverse aspects of the destination's cultural | 0.756 |  |  |  |  |
| Tourist satisfaction |  |  | 0.875 | 72.354 | 0.633 | 0.873 |
| TS1 | Generally satisfied | 0.742 |  |  |  |  |
| TS2 | All expectations are fulfilled | 0.811 |  |  |  |  |
| TS3 | Have fun | 0.786 |  |  |  |  |
| TS4 | Time and money spent are satisfied | 0.839 |  |  |  |  |
| Tourist loyalty |  |  | 0.84 | 75.835 | 0.647 | 0.845 |
| TL1 | Stay here again | 0.696 |  |  |  |  |
| TL2 | Others are recommended to travel here | 0.898 |  |  |  |  |
| TL3 | Share travel updates via social media (WeChat, QQ, etc.) | 0.806 |  |  |  |  |

Note(s): SFL=Standardized Factor Loading; VCC=variance cumulative contribution; α=Cronbach's alpha.

Table 6. Results of the perceived value discriminant validity test.

| **Model** | **χ2** | **df** | **χ2/df** | **NFI** | **CFI** | **RMSEA** | **Model Comparison** | **∆χ2** | **∆df** |
| --- | --- | --- | --- | --- | --- | --- | --- | --- | --- |
| Three factor model | 65.807 | 51 | 1.29 | 0.978 | 0.995 | 0.028 |  |  |  |
| Two factor model Ⅰ | 168.306 | 53 | 3.176 | 0.944 | 0.961 | 0.112 | 2 VS 1 | 102.499*** | 2 |
| Two factor model Ⅱ | 120.654 | 53 | 1.937 | 0.966 | 0.983 | 0.05 | 3 VS 1 | 54.847*** | 2 |
| Single factor model | 244.054 | 54 | 4.52 | 0.918 | 0.935 | 0.096 | 4 VS 1 | 178.247*** | 3 |

Note(s): ***p < 0.001, Two factor model Ⅰ=F1+F2, F3; Two factor model Ⅱ=F2+F3, F1; Single factor model= F1+F2+F3; F1: cultural ontology distortion; F2: cultural representation distortion; F3: cultural construction distortion.

Table 7. Results of the perceived value discriminant validity test.

| **Variable** | **Quality value** | **Emotional value** | **Price value** | **Novelty value** |
| --- | --- | --- | --- | --- |
| Quality value | **0.780** |  |  |  |
| Emotional value | 0.708** | **0.812** |  |  |
| Price value | 0.674** | 0.715** | **0.788** |  |
| Novelty value | 0.524** | 0.677** | 0.664** | **0.760** |

Note(s): **p < 0.01.

Table 8. Results of model direct path relationship test.

| **Hypotheses** | **β** | **T-value** | **P-value** | **Supported** |
| --- | --- | --- | --- | --- |
| H1 CDR → PEV | -0.544 | -7.649 | *** | Yes |
| H1a CDR → TS | 0.102 | 1.998 | 0.046 | Yes |
| H1b CDR → TL | -0.055 | -1.142 | 0.254 | No |
| H2a PEV → TS | 0.909 | 9.819 | *** | Yes |
| H2b PEV →TL | 0.072 | 0.634 | 0.526 | No |
| H3a TS →TL | 0.804 | 7.170 | *** | Yes |

Note(s): CDR=Cultural distortion risk; PEV=Perceived value; TS=Tourist satisfaction; TL=Tourist loyalty.

Table 9. Results of model indirect path relationship test.

| **Hypotheses** | **Effect** | **β** | **95% confidence interval** | | **P** |
| --- | --- | --- | --- | --- | --- |
|  |  |  | **LB** | **UB** |  |
| CDR→TL | Total effcet | -0.409 | -0.516 | -0.297 | 0.000 |
|  | Direct effect | -0.055 | -0.163 | 0.042 | 0.276 |
|  | CDR→TS→TL | 0.082 | -0.008 | 0.192 | 0.071 |
|  | CDR→PEV→TL | -0.039 | -0.186 | 0.118 | 0.608 |
|  | CDR→PEV→TS→TL | -0.397 | -0.579 | -0.258 | 0.000 |
|  | Total indirect effect | -0.354 | -0.474 | -0.231 | 0.000 |

Note(s): CDR=Cultural distortion risk; TL=Tourist loyalty；TS=Tourist satisfaction; PV=Perceived value.
