# Supplementary material for: Cultural distortion risk and tourist loyalty at silk road heritage: The mediating roles of perceived value and satisfaction
Source: PLoS One. 2025 Nov 5;20(11):e0335476. doi: 10.1371/journal.pone.0335476 (PMC12588480; doi:10.1371/journal.pone.0335476)
Supplement: S1 File — (DOCX) [file pone.0335476.s004.docx]

# Questionnaire on Cultural Heritage Tourism of the Gansu Section of the Silk Road

**Dear Sir/Madam：**
Hello！
     We are the Tourism Risk Research Group of Northwest Normal University. This questionnaire is about cultural heritage tourism in the Gansu section of the Silk Road. The questionnaire is anonymous and the information obtained is only used for academic research and strictly confidential. There are no correct or incorrect answers in this questionnaire. Please fill it out carefully according to the actual situation. Thank you very much for taking the time to fill out this survey questionnaire. Wishing you a happy life!

Please mark a "√" in the corresponding position according to the actual situation.

| **Part 1 Questionnaire Question Items** | | **Strongly disagree** | **disagree** | **generally** | **agree** | **strongly agree** |
| --- | --- | --- | --- | --- | --- | --- |
| 1、 | I am concerned that the original culture of heritage tourism destinations may deteriorate. | 1 | 2 | 3 | 4 | 5 |
| 2、 | I'm worried that the customs and traditions of heritage tourism destinations may be lost. | 1 | 2 | 3 | 4 | 5 |
| 3、 | I am concerned that the cultural heritage of heritage tourism destinations may be lost. | 1 | 2 | 3 | 4 | 5 |
| 4、 | I'm worried that the heritage site has deviated from its original historical and cultural heritage. | 1 | 2 | 3 | 4 | 5 |
| 5、 | I am concerned that the presentation of heritage tourism destinations may not match the original historical style. | 1 | 2 | 3 | 4 | 5 |
| 6、 | I am concerned about the disconnect between the display of heritage tourism destinations and the essence of local culture. | 1 | 2 | 3 | 4 | 5 |
| 7、 | I am concerned about the lack of cultural display methods in heritage tourism destinations. | 1 | 2 | 3 | 4 | 5 |
| 8、 | I'm worried that heritage tourism destinations may have cultural products that are monotonous. | 1 | 2 | 3 | 4 | 5 |
| 9、 | I am concerned that people may not be able to recognize the cultural uniqueness of heritage tourism destinations. | 1 | 2 | 3 | 4 | 5 |
| 10、 | I am concerned that people may find it difficult to understand the cultural knowledge of heritage tourism destinations. | 1 | 2 | 3 | 4 | 5 |
| 11、 | I am concerned that people's cultural awareness of cultural heritage tourism destinations may be vague. | 1 | 2 | 3 | 4 | 5 |
| 12、 | I am concerned that people have a weak cultural impression of cultural heritage tourism destinations. | 1 | 2 | 3 | 4 | 5 |
| 13、 | Maintained uniform quality standards across multiple iterations. | 1 | 2 | 3 | 4 | 5 |
| 14、 | The scenic area is well done. | 1 | 2 | 3 | 4 | 5 |
| 15、 | Met or exceeded industry-recognized quality thresholds. | 1 | 2 | 3 | 4 | 5 |
| 16、 | The efficiency of tourism services is high. | 1 | 2 | 3 | 4 | 5 |
| 17、 | A sustained state of psychological fulfillment and physiological comfort. | 1 | 2 | 3 | 4 | 5 |
| 18、 | Stimulated a sense of exhilaration. | 1 | 2 | 3 | 4 | 5 |
| 19、 | Made me related. | 1 | 2 | 3 | 4 | 5 |
| 20、 | Made me feel happy. | 1 | 2 | 3 | 4 | 5 |
| 21、 | Delivered equitable returns on monetary investment relative. | 1 | 2 | 3 | 4 | 5 |
| 22、 | Optimal alignment with market expectations for comparable service tiers. | 1 | 2 | 3 | 4 | 5 |
| 23、 | Good one for the price paid. | 1 | 2 | 3 | 4 | 5 |
| 24、 | The pricing structure adheres to affordability thresholds. | 1 | 2 | 3 | 4 | 5 |
| 25、 | This trip can gain social recognition from others. | 1 | 2 | 3 | 4 | 5 |
| 26、 | This trip can improve others' perception and evaluation of my personal image. | 1 | 2 | 3 | 4 | 5 |
| 27、 | This trip can increase others' acceptance of me. | 1 | 2 | 3 | 4 | 5 |
| 28、 | This trip can leave a positive impression on others. | 1 | 2 | 3 | 4 | 5 |
| 29、 | Stimulated my sense of exploration and discovery. | 1 | 2 | 3 | 4 | 5 |
| 30、 | Addressed my desire for novel cultural encounters. | 1 | 2 | 3 | 4 | 5 |
| 31、 | Provided genuine immersion in local traditions. | 1 | 2 | 3 | 4 | 5 |
| 32、 | Enabled comprehensive engagement with diverse aspects of the destination's cultural. | 1 | 2 | 3 | 4 | 5 |
| 33、 | Generally satisfied. | 1 | 2 | 3 | 4 | 5 |
| 34、 | All expectations are fulfilled. | 1 | 2 | 3 | 4 | 5 |
| 35、 | Have fun. | 1 | 2 | 3 | 4 | 5 |
| 36、 | Time and money spent are satisfied. | 1 | 2 | 3 | 4 | 5 |
| 37、 | Stay here again. | 1 | 2 | 3 | 4 | 5 |
| 38、 | Others are recommended to travel here. | 1 | 2 | 3 | 4 | 5 |
| 39、 | Share travel updates via social media (WeChat, QQ, etc.) | 1 | 2 | 3 | 4 | 5 |
| 40、 | Stimulated my sense of exploration and discovery. | 1 | 2 | 3 | 4 | 5 |

**Part 2 Personal Information**

1. Gender： A male B female
2. 2、Age： A 18-29 B 30-39 C 40-49 D 50 and above

3、Education： A Senior middle school and below B Junior High C Senior High D University

E Master or Doctor

4、Monthly income： A 3000 and below B 3001-5000 C 5001-10000 D 10001-20000 E 20001 and above

5、Occupation： A Professional B Company employee C Manufacturer/Technician D Service employee E Independent businessman F Government official/Teacher G Student H Other

6、Have you had any other experiences of Silk Road heritage tourism： A Yes B NO
