# Supplementary material for: Cultural distortion risk and tourist loyalty at silk road heritage: The mediating roles of perceived value and satisfaction
Source: PLoS One. 2025 Nov 5;20(11):e0335476. doi: 10.1371/journal.pone.0335476 (PMC12588480; doi:10.1371/journal.pone.0335476)
Supplement: S2 File — (PDF) [file pone.0335476.s005.pdf]

Survey Questionnaire Informed Consent Form

Dear Visitor,

Thank you for participating in this academic survey! To ensure your right to informed consent and voluntary participation, please read the following information carefully before completing the questionnaire:

1. Research Purpose

This study is conducted by the College of Tourism, Northwest Normal University. It aims to analyze tourists' perceived value and satisfaction, assess the impact of cultural distortion risks in the tourism activities of the Maijishan Grottoes on visitor loyalty, and provide a basis for developing scientific conservation strategies.

2. Survey Organization

Host Institution: College of Tourism, Northwest Normal University

3. Your Rights

You are free to choose whether to participate. Refusal will not affect any of your rights at the scenic site; The questionnaire is for academic research only and does not collect personal information such as your name or ID number; All data will be encrypted and stored by the research team, used solely for this study, and will not be disclosed or used for commercial purposes; You may stop filling out the questionnaire at any time, even after starting.

4. Questionnaire Content

The survey includes the following sections (approximately 5-8 minutes):

Basic Information: Age, occupation, gender (no private details required).

Risk Perception: Your understanding of cultural distortion risks at the Maijishan Grottoes.

Perceived Value: Your perceptions of quality, price, emotional experience, and novelty.

Satisfaction: Your satisfaction level with the Maijishan Grottoes.

Loyalty: Your loyalty toward the Maijishan Grottoes.

5. Research Ethics

This study adheres to academic standards and guarantees: The questionnaire does not involve any sensitive or private issues; Data collection and analysis processes comply with principles of academic integrity; All responses will be anonymized, and participation is entirely voluntary.

6. Contact Information

For any questions or feedback regarding this survey, please contact:

Principal Investigator: Associate Professor Kejun Wu

Email: wkj966516@nwnu.edu.cn

Please confirm by checking the boxes below:

□ I have read and understood the above information, and voluntarily agree to participate in this survey.

□ I understand that I may withdraw from the questionnaire at any time, and that the data will be used solely for academic research purposes.

Researcher's Statement of Disclosure

I have informed the participant of the study background, the research institution, his/her rights, and the questionnaire content related to the project named [Project Title]. The participant has been given sufficient time to read the informed consent form, discuss it with others, and have all study-related questions answered. I have provided the participant with contact information should any issues arise. I have also informed the participant (or his/her legal representative) that he/she may withdraw from the study at any time without giving any reason.

西北师范大学
旅游学院
